# Regulation of the WNT-CTNNB1 signaling pathway by severe fever with thrombocytopenia syndrome virus in a cap-snatching manner

**DOI:** 10.1128/mbio.01688-23

**Published:** 2023-10-26

**Authors:** Xia-Ming Jiang, Qi-Lin Xin, Kai Liu, Xue-Fang Peng, Shuo Han, Ling-Yu Zhang, Wei Liu, Geng-Fu Xiao, Hao Li, Lei-Ke Zhang

**Affiliations:** 1 State Key Laboratory of Virology, Wuhan Institute of Virology, Center for Biosafety Mega-Science, Chinese Academy of Sciences, Wuhan, Hubei, China; 2 University of Chinese Academy of Sciences, Beijing, China; 3 University of Lyon, INRAE, EPHE, IVPC, Lyon, France; 4 School of Pharmaceutical Engineering, Shenyang Pharmaceutical University, Shenyang, China; 5 National Engineering Research Center for the Emergency Drug, Beijing Institute of Pharmacology and Toxicology, Beijing, China; 6 State Key Laboratory of Pathogen and Biosecurity, Beijing Institute of Microbiology and Epidemiology, Beijing, China; 7 Hubei Jiangxia Laboratory, Wuhan, China; Washington University in St. Louis School of Medicine, St. Louis, Missouri, USA; Johns Hopkins University Bloomberg School of Public Health, Baltimore, Maryland, USA

**Keywords:** sNSVs, WNT-CTNNB1 signaling pathway, cap-snatching, severe fever with thrombocytopenia syndrome virus

## Abstract

The segmented negative-strand RNA viruses (sNSVs) include highly pathogenic human and animal viruses such as Lassa virus (LASV), severe fever with thrombocytopenia syndrome virus (SFTSV), and influenza A virus (IAV). One of the conserved mechanisms at the stage of genome transcription of sNSVs is the cap-snatching process, providing druggable targets for the development of antivirals. SFTSV is an emerging tick-borne sNSV that causes severe hemorrhagic fever with a high fatality rate of 12%–50%. Here, we determined the correlation between death outcome and downregulation of the WNT-CTNNB1 signaling pathway through transcriptomic analysis of blood samples collected from SFTS patients. We further demonstrated that SFTSV affected this pathway by downregulating the mRNA levels of a series of pathway-related genes, including *CTNNB1*. Loss-of-function mutations or inhibitors targeting SFTSV cap-snatching activity effectively alleviated the inhibition of the WNT-CTNNB1 signaling pathway. Exogenous activation of the WNT-CTNNB1 signaling pathway enhanced SFTSV replication, while inhibition of this pathway reduced SFTSV replication. Treatment with a WNT-CTNNB1 signaling pathway inhibitor attenuated viral replication and decreased fatality in mice. Notably, downregulation of the WNT-CTNNB1 signaling pathway was also observed for other sNSVs, including LASV and IAV. These results suggested that RNAs related to the WNT-CTNNB1 signaling pathway might be utilized as a primer “pool” in a cap-snatching manner for viral transcription, which provides effective targets for the development of broad-spectrum antivirals against sNSVs.

**IMPORTANCE:**

One of the conserved mechanisms at the stage of genome transcription of segmented negative-strand RNA viruses (sNSVs) is the cap-snatching process, which is vital for sNSVs transcription and provides drugable targets for the development of antivirals. However, the specificity of RNAs snatched by sNSV is still unclear. By transcriptomics analysis of whole blood samples from SFTS patients, we found WNT-CTNNB1 signaling pathway was regulated according to the course of the disease. We then demonstrated that L protein of severe fever with thrombocytopenia syndrome virus (SFTSV) could interact with mRNAs of WNT-CTNNB1 signaling pathway-related gene, thus affecting WNT-CTNNB1 signaling pathway through its cap-snatching activity. Activation of WNT-CTNNB1 signaling pathway enhanced SFTSV replication, while inhibition of this pathway decreased SFTSV replication *in vitro* and *in vivo*. These findings suggest that WNT-associated genes may be the substrate for SFTSV “cap-snatching”, and indicate a conserved sNSVs replication mechanism involving WNT-CTNNB1 signaling.

## INTRODUCTION

The segmented negative-strand RNA viruses (sNSVs) include the families *Arenaviridae*, *Peribunyaviridae*, and *Orthomyxoviridae* (1). Highly pathogenic human and animal viruses such as Lassa virus (LASV), severe fever with thrombocytopenia syndrome virus (SFTSV), and influenza A virus (IAV) belong to these families. Approximately 100,000–300,000 cases of LASV infection are reported in West Africa each year, with an overall case fatality rate (CFR) of 1%–2% (2). SFTSV is endemic in eastern and southeastern Asia and can cause severe hemorrhagic fever, accompanied by encephalitis, disseminated intravascular coagulation, and multiple organ failure, with a high CFR of 12%–50% (3, 4). IAV infection is responsible for an estimated 500,000 deaths and up to 5 million cases of severe respiratory illness each year (5). Currently, effective antiviral therapeutic strategies are still limited or lacking for these sNSVs. There is a pressing need to better understand the pathogenesis of sNSVs and identify therapeutic strategies to ameliorate disease outcomes.

At the stage of genome transcription of sNSVs, viral mRNA synthesis is primed using short 5′ methyl-7-guanosine (m^7^G)-capped RNA sequences, which are cleaved from host RNA polymerase II transcripts by the viral polymerase complex in a process known as “cap-snatching” (6). This mechanism involves a cap-binding (CapB) domain and an endonuclease (Endo) domain of the polymerase complex (7). The viral RNA-dependent RNA polymerase (RdRp) domain utilizes the “stolen” cellular capped RNA fragments as primers to initiate viral transcription, which generates viral mRNAs with a host RNA-derived sequence at the 5′-end (8) and creates viral transcripts that are genetic hybrids of host and viral sequences. A recent study indicated that translation from host-derived upstream start codons in chimeric host-viral transcripts and corresponding chimeric proteins made in IAV-infected cells can generate T-cell responses and contribute to virulence (1). However, host-derived sequences are highly diverse, and the consequences of this process on host cells still need to be clarified (8–10).

The WNT-CTNNB1 signaling pathway, or canonical WNT pathway, is an evolutionarily conserved signaling cascade that involves activation of the transcriptional coactivator CTNNB1/β-catenin (11). CTNNB1-dependent WNT signaling pathways have crucial roles in the regulation of diverse cell behaviors, including cell fate, proliferation, survival, differentiation, migration, and polarity (12). A central event in this signaling cascade is the regulated proteolytic turnover of the transcriptional coactivator CTNNB1. In the “OFF” state, free cytoplasmic CTNNB1 is rapidly sequestered by a cytoplasmic “destruction complex,” and then targeted for ubiquitination and proteasomal degradation. In the “ON” state, the binding of WNTs to frizzled and LRP5 or LRP6 coreceptors transduces a signal across the plasma membrane that results in the activation of the dishevelled (DVL) protein. Activated DVL inhibits the destruction complex, resulting in the accumulation of CTNNB1, which promotes the transcription of genes related to proliferation and survival by acting as a coactivator for the T-cell factor/lymphoid enhancer factor (TCF/LEF) family of transcription factors (13). The importance of positive and negative feedback loops in the dynamic regulation of developmental signaling has been highlighted (14). Several WNT pathway components can act as activators or potentiate WNT signaling, while to prevent excessive signaling, WNT-induced responses are also balanced by numerous negative feedback regulators (12).

The WNT-CTNNB1 signaling pathway has also been implicated in the life cycles of various viruses, including influenza virus (15, 16), Rift Valley fever virus (17), Zika virus (18), human immunodeficiency virus (19, 20), and human cytomegalovirus (21), but the molecular mechanism remains unclear. Studies have reported that pathogenic viruses could manipulate the WNT pathway for productive infection, and progress has been made in the development of WNT inhibitors for cancer treatments, suggesting that inhibition of the WNT pathway might serve as a new avenue for developing host-targeted antiviral therapeutics (10). To date, there are various ways to inhibit WNT signaling *in vitro* and *in vivo*. One option is applying RNAi targeting various components of the pathway, such as DVL2 (22), and a variety of small molecules have been shown to either inhibit or activate WNT signaling to various degrees (23–26).

By transcriptomics analysis of whole blood samples from SFTS patients, we found that multiple signaling pathways were regulated along with the course of the disease, and one of the most significantly regulated pathways was the WNT-CTNNB1 signaling pathway. We then demonstrated that SFTSV L protein could interact with mRNAs of WNT-CTNNB1 signaling pathway-related genes and regulate their expression through its cap-snatching manner, thus affecting the WNT-CTNNB1 signaling pathway. We further found that WNT-associated RNAs may be substrates for cap-snatching of SFTSV or IAV, suggesting a conserved sNSV replication mechanism involving the WNT-CTNNB1 signaling pathway. This mechanism of SFTSV replication, which might exist widely in sNSVs (15, 17), could provide a theoretical basis for the development of effective broad-spectrum host-directed antiviral therapeutics.

## RESULTS

### Transcriptome analyses revealed inhibition of disease-associated pathways during SFTSV infection

**Fig 1:**
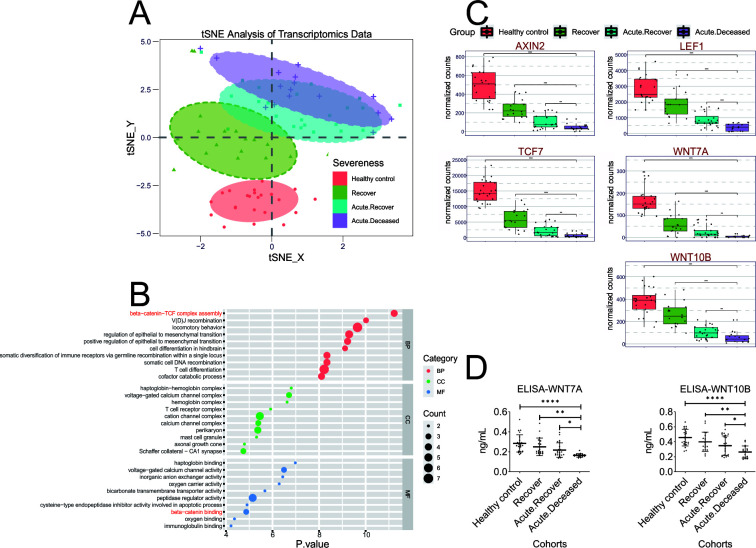
Transcriptomic analyses reveal inhibition of the WNT-CTNNB1 signaling pathway by SFTSV. (A–C) Transcriptomic analysis of whole blood isolated from four groups of patients: healthy controls, recovered patients, acute recovered patients, and acute deceased patients. (A) *t*-SNE plot of transcriptomic data of the four groups. (B) Gene ontology (GO) analysis of downregulated genes between the Acute.Recover and Acute.Deceased groups highlighted WNT-CTNNB1 signaling pathway. Subsets of downregulated genes with fold change of expression (|log_2_FC| ≥ 1) and *P* value (*P* < 0.05) in differentially expressed genes (DEGs) (Acute.Deceased/Acute.Recover) were screened for GO analysis. BP, biological process; CC, cellular component; and MF, molecular function. GO terms of interest were highlighted in red. (C) Transcriptomic analyses show that the expression of WNT-CTNNB1 signaling pathway-related genes was significantly downregulated by SFTSV. These five genes were selected from the gene set enriched to the WNT-CTNNB1 signaling pathway, including AXIN2, TCF7, LEF1, WNT7A, and WNT10B. (D) Secreted Wnt7a and Wnt10b levels in serum of the four groups of patients decreased as the disease progressed. Concentrations of secreted Wnt7a and Wnt10b in the serum samples were determined using the human protein Wnt-7a (WNT7A) and Wnt-10b (WNT10B) ELISA (enzyme-linked immunosorbent assay) kits (ELISA-WNT7A and ELISA-WNT10B). Comparison of mean values (C and D) was analyzed by one-way ANOVA (analysis of variance) analyses. (D) The experiment was performed with three technical replicates.

To investigate the pathogenesis mechanisms of SFTSV infection, we performed a transcriptomic analysis of blood samples collected from 81 peripheral blood samples from 40 SFTS patients and 23 healthy donors. For SFTS patients, 58 peripheral blood samples were divided into three groups based on clinical status: “Recover” indicated samples (*n* = 19) from SFTS patients in convalescence who had recovered from acute virus infection; “Acute.Recover” indicated samples from SFTS patients (*n* = 22) during acute virus infection who eventually recovered from the disease; and “Acute.Deceased” indicated samples (*n* = 17) from SFTS patients during acute virus infection who eventually died of SFTSV infection. Transcriptomic analysis was performed on the blood samples to determine differences and consistency across clinical sample groups, and *t*-distributed stochastic neighbor embedding (*t*-SNE) analysis showed that the targeted transcriptomic profiles distinguished patients from distinct groups (Fig. 1A) (27). This technique maps a set of high-dimensional points to two dimensions, such that ideally, close neighbors remain close and distant points remain distant (28). In essence, the distance within the *t*-SNE results indicates the extent of similarity in alterations of the data’s structure. In our findings, this corresponds to the magnitude of variation in the mRNA of diverse patients afflicted with SFTSV.

The blood samples of the “Acute.Recover” and “Acute.Deceased” cohorts were both collected during the acute infection phase; thus, they were not completely separated, and differences in transcript levels between these two groups may explain different outcomes for patients in the acute phase. To identify factors contributing to an SFTS fatal infection outcome, we analyzed the differentially regulated genes between the two acute infection patient groups. Differentially expressed genes (DEGs) with |log_2_FC| ≥ 1 and *P* value <0.05 were selected for further analysis. Gene ontology (GO) analysis of the DEGs showed more than 200 GO terms (*P* < 0.01) were identified, specifically the downregulated categories were related to “beta-catenin-TCF complex assembly,” “T cell differentiation,” and “beta-catenin binding ” when comparing the two acute infection patient groups (Fig. 1B). “Beta-catenin-TCF complex” (*P* < 0.0001) and “beta-catenin binding” (*P* = 0.0077) belongs to the WNT-CTNNB1 signaling pathway, which has been reported to play roles in cell proliferation and differentiation (29, 30). The WNT-CTNNB1 signaling pathway-related genes in the gene set were further analyzed, including AXIN2, LEF1, TCF7, WNT7A, and WNT10B, and we found that these genes decreased with the severity of the patients (Fig. 1C). To further verify the results of transcriptome data analysis, we detected the concentrations of secreted Wnt7a and Wnt10b in the serum samples of the above four groups using the human protein Wnt-7a (WNT7A) and Wnt-10b (WNT10B) enzyme-linked immunosorbent assay (ELISA) kits and found that there were differences between the cohorts, and the secreted protein level of “Acute.Deceased” was significantly lower than “Acute.Recover” (Fig. 1D). In addition, through *ex vivo* infection of peripheral blood mononuclear cells (PBMCs) collected from healthy donors, SFTSV inhibited the transcription levels of WNT7A and WNT10B (Fig. S1). These results indicated a potential role of viral regulation of the WNT-CTNNB1 signaling pathway in SFTS fatality.

### The L protein of SFTSV inhibits the WNT-CTNNB1 signaling pathway

We then explored the effects of SFTSV infection on the WNT-CTNNB1 signaling pathway in human embryonic kidney (HEK) 293T cells, a commonly used cell line to study the WNT-CTNNB1 signaling pathway (31) and a permissive cell line for SFTSV infection. Reported studies have employed activators to maintain the WNT-CTNNB1 signaling pathway in an activated state in HEK 293T cells (12, 32). Here, a reporter system for the WNT-CTNNB1 signaling pathway, namely, TOPFlash (15, 33, 34), was established. HEK 293T cells were co-transfected with a TOPFlash reporter plasmid, which contains three copies of TCF binding sites upstream of the firefly luciferase gene and a pRL-TK internal control luciferase reporter plasmid for normalization, which contains Renilla luciferase gene. Briefly, HEK 293T cells were transfected with TOPFlash and pRL-TK plasmids and treated with 6-bromoindirubin-3′-oxime (BIO) and LiCl, two reported activators of the WNT-CTNNB1 signaling pathway (35, 36). BIO activated the TOPFlash reporter system more significantly (Fig. S2A). Then, we detected the intracellular protein level of CTNNB1 and found that BIO treatment resulted in more significant accumulation of CTNNB1 (Fig. S2B); thus, BIO was chosen as the activator for the WNT-CTNNB1 signaling pathway for the following study.

**Fig 2:**
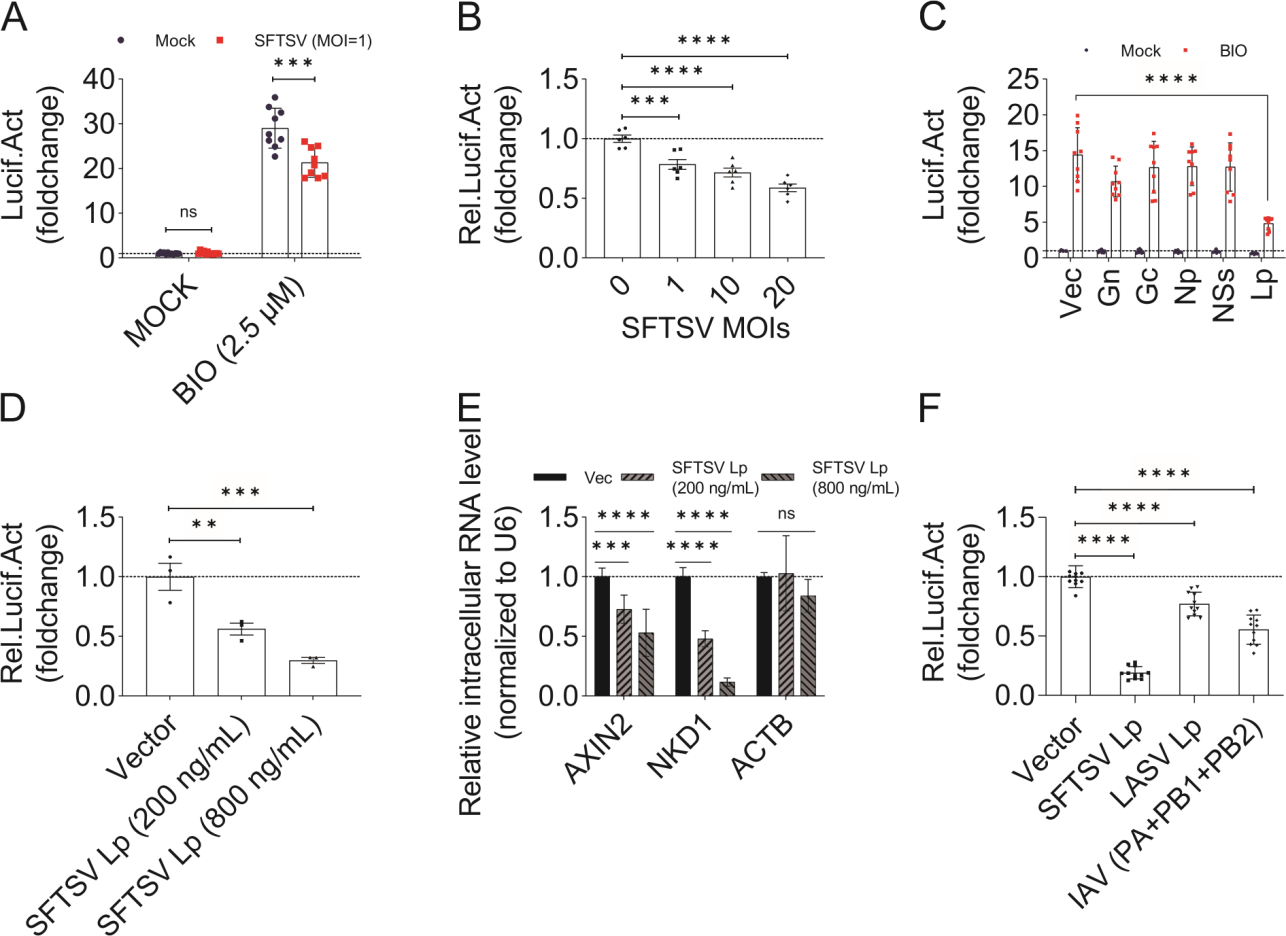
SFTSV infection inhibits the activated WNT-CTNNB1 signaling pathway via its L protein. (A and B) SFTSV infection inhibited activator-triggered TOPFlash activity. In 48-well plates, HEK 293T cells were transfected with TOPFlash reporter plasmids (50 ng/well) and pRL-TK internal control luciferase reporter plasmid (50 ng/well). After 24 h, cells that were nonactivated (mock) or activated (BIO, 2.5 µM) for 6 h were infected with SFTSV at an MOI of 1 (A) or the indicated MOIs (1, 10, 20) (B). Another 18 h under-treated (mock or BIO) and infected, cells were lysed and subjected to luciferase assays to measure TOPFlash activity. (C and D) SFTSV L protein inhibited TOPFlash activity. (C) SFTSV L protein inhibited TOPFlash activity. In 48-well plates, HEK 293T cells were transfected with TOPFlash reporter plasmids (50 ng/well), pRL-TK internal control luciferase reporter plasmids (50 ng/well) and pCAGGS plasmids encoding SFTSV proteins (Gn, Gc, NSs, N protein, and L protein) with a C-terminal Twin-Strep-tag (called “pCAAGS-SFTSV Gn/Gc/NSs/Np/Lp-TS") or a pCAGGS vector plasmid (150 ng/well) expressing only the Twin-Strep-tag as a control. At 24-h post-transfection, cells were treated with BIO (2.5 µM) or mock-treated. After 24 h, cells were lysed and subjected to luciferase assays to measure TOPFlash activity. Vec, pCAGGS vector plasmid; Gn/Gc/Np/NSs/Lp, pCAGGS plasmid encoding SFTSV Gn, Gc, NSs, N, or L protein. (D) SFTSV L protein inhibited TOPFlash activity in a dose-dependent manner at the indicated concentration. In 48-well plates, HEK 293T cells were transfected with TOPFlash reporter plasmids (50 ng/well), pRL-TK internal control luciferase reporter plasmids (50 ng/well) and pCAGGS plasmids encoding SFTSV L protein or vector. The transfection amounts of pCAGGS plasmids encoding SFTSV L protein were 37.5 or 150 ng/well (200 or 800 ng/mL), and supplemented with vector. At 24-h post-transfection, cells were treated with BIO (2.5 µM) or mock-treated. After 24 h, cells were lysed and subjected to luciferase assays to measure TOPFlash activity. Vector, Vec, pCAGGS vector plasmid; SFTSV Lp, pCAGGS plasmid encoding SFTSV L protein. (E) SFTSV L protein inhibited the transcription of downstream genes in the WNT-CTNNB1 signaling pathway. In six-well plates, HEK 293T cells were transfected with pCAGGS plasmids encoding SFTSV L protein or vector. The transfection amounts of pCAGGS plasmids encoding SFTSV L protein were 400 or 1,600 ng/well (200 or 800 ng/mL), and supplemented with vector. At 24-h post-transfection, cells were treated with BIO (5 µM). After 6 h, cells were collected and intracellular RNA was extracted. Quantitative real-time PCR (qRT-PCR) analysis of BIO-induced activation of target genes *AXIN2*, *NKD1* and, as a control, *ACTB* normalized to *U6*. Vec, pCAGGS vector plasmid; SFTSV Lp, pCAGGS plasmid encoding SFTSV L protein. (F) LASV L protein and IAV L protein (PA, PB1, and PB2 complex) inhibited TOPFlash activity. In 48-well plates, HEK 293T cells were transfected with TOPFlash reporter plasmids (50 ng/well), pRL-TK internal control luciferase reporter plasmids (50 ng/well), and pCAGGS plasmids encoding viral proteins (SFTSV/LASV/IAV L protein or polymerase complex) with a C-terminal Twin-Strep-tag (called “pCAAGS-SFTSV Lp-TS", “pCAGGS-LASV Lp-TS,” and “pCAGGS-PR8 PA/PB1/PB2-TS”) or a pCAGGS vector plasmid (150 ng/well) expressing only the Twin-Strep-tag as a control. At 24-h post-transfection, cells were treated with BIO (2.5 µM). After 24 h, cells were lysed and subjected to luciferase assays to measure TOPFlash activity. Vector, pCAGGS plasmid vector; SFTSV Lp, pCAGGS plasmid encoding SFTSV L protein; LASV Lp, pCAGGS plasmid encoding LASV L protein; IAV (PA + PB1 + PB2), pCAGGS plasmid encoding IAV PA, PB1, or PB2 protein. Comparison of mean values (A–F) between two groups was analyzed by one-way ANOVA or two-way ANOVA analyses. (A–F) All experiments were performed with three independent replicates and any independent replicates had at least two technical replicates.

HEK 293T cells transfected with the TOPFlash system were treated with BIO for 6 h, and then infected with SFTSV at an MOI (multiplicity of infection) of 1 for more 18 h, subjected to luciferase assays (Fig. 2A). We found that SFTSV infection inhibited BIO-triggered TOPFlash activity in a dose-dependent manner (Fig. 2B). The SFTSV genome encodes L protein, GP (Gn and Gc), N protein, and NSs. To investigate which viral protein inhibited the WNT-CTNNB1 signaling pathway, HEK 293T cells were transfected with the TOPFlash system and plasmids encoding SFTSV proteins with a C-terminal Twin-Strep-tag (Fig. S3) and then treated with BIO. We found that SFTSV L protein, but not other viral proteins, significantly inhibited TOPFlash activity (Fig. 2C). We further found that SFTSV L protein inhibited TOPFlash activity and transcription of downstream genes (*AXIN2* and *NKD1*) in the WNT-CTNNB1 signaling pathway in a dose-dependent manner (Fig. 2D and E). In addition, we found that both LASV L protein and IAV polymerase complex (PA, PB1, and PB2; Fig. S4) had similar inhibitory effects on TOPFlash activity (Fig. 2F). Out of these, the SFTSV L protein exhibited the most potent inhibitory effect at 80% ± 5%, followed by the IAV polymerase complex at 50% ± 10%, and then the LASV L protein at about 25% ± 10%. This outcome suggests that the suppression of the WNT-CTNNB1 signaling pathway might be a shared feature among sNSVs, with SFTSV’s L protein demonstrating relatively stronger inhibition compared to that of IAV or LASV.

### Inhibition of the WNT-CTNNB1 signaling pathway by L protein is associated with its endonuclease activity and alleviated by a CapB inhibitor

**Fig 3:**
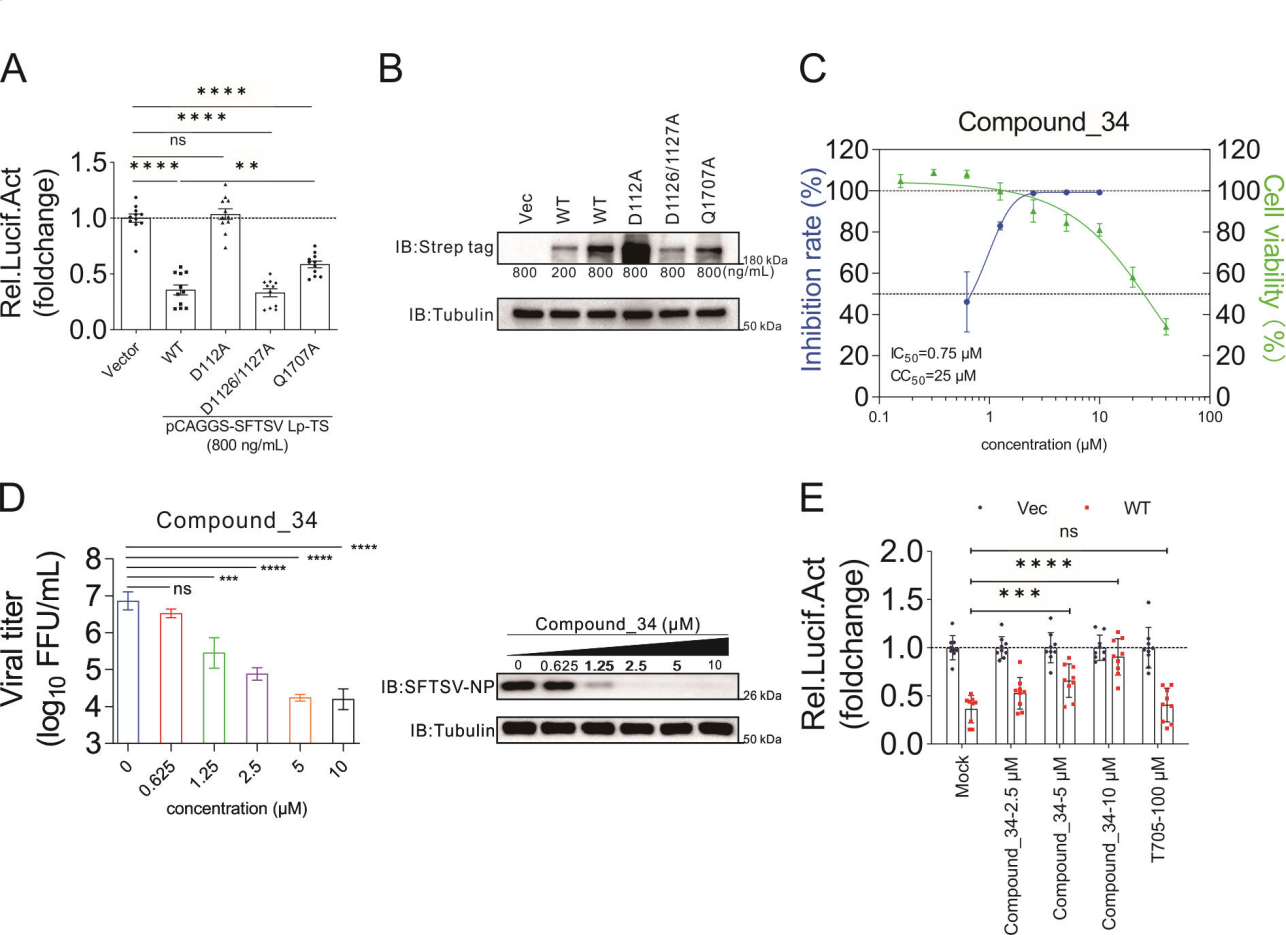
Inhibition of the WNT-CTNNB1 signaling pathway by L protein is associated with its cap-snatching activity. (A and B) The loss of endonuclease activity of SFTSV L protein alleviated its inhibitory effect on TOPFlash activity. (A) In 48-well plates, HEK 293T cells were transfected with TOPFlash reporter plasmids (50 ng/well), pRL-TK internal control luciferase reporter plasmids (50 ng/well), and pCAGGS plasmids encoding SFTSV L protein (wild type or mutants) or vector (150 ng/well). At 24-h post-transfection, cells were untreated (mock) or treated (BIO, 2.5 µM). After 24 h, cells were lysed and subjected to luciferase assays to measure TOPFlash activity. (B) Western blot analysis was also conducted to show SFTSV L protein (wild type or mutants) could be successfully expressed. In six-well plate, HEK 293T cells were transfected with plasmids (wild type, mutants, or vector) at the indicated concentrations. After 24 h, the cells were lysed and subjected to Western blot analysis. SFTSV L proteins (wild type or mutants) were detected by an anti-Strep tag antibody. Tubulin was also detected as a control for each lane. (C and D) Compound_34 can effectively inhibit SFTSV replication. In 24-well plates, HEK 293T cells were pre-infected with SFTSV at an MOI of 0.1 for 1 h. The cells were treated with a series of concentrations of Compound_34 as indicated. At 48 h post-infection, intracellular RNA was extracted (C), and cells and supernatants were collected and subjected to Western blot analysis and virus titers determination (D). (C) CC_50_ and IC_50_ of Compound_34 for SFTSV in HEK 293T cells. The intracellular level of SFTSV RNA was measured with quantitative real-time PCR (qRT-PCR). Intracellular levels of *GAPDH* mRNA were used as the endogenous control. The cytotoxicity of Compound_34 in HEK 293T cells was also determined by using CCK8 assay. The left and right *Y*-axis of the graphs represented inhibition rate (%) and cytotoxicity viability (%), respectively, of Compound_34. The four-parameter dose–response curve was fitted using the nonlinear regression method and values were calculated in the software Prism 9.0. (D) Supernatants of each concentration of compound treatment were collected for viral titer determination. Detailed methods and procedures for viral FFU determination are in the Materials and Methods (left). SFTSV N protein was also detected by a specific antiserum, serving as a maker to reflect the replication level of the virus. Tubulin was also detected as a control for each lane. The concentration of the compound was shown above the band, and the triangle showed the trend of increasing concentration (right). (E) SFTSV L protein CapB inhibitor Compound_34 alleviated the inhibitory effect on TOPFlash activity of SFTSV L protein. In 48-well plates, HEK 293T cells were transfected with TOPFlash reporter plasmids (50 ng/well), pRL-TK internal control luciferase reporter plasmids (50 ng/well) and pCAGGS plasmids encoding SFTSV L protein or vector (150 ng/well). At 24-h post-transfection, the cells were activated (BIO, 2.5 µM) and treated with Compound_34 or T-705 (favipiravir) at the indicated concentrations. After 24 h, cells were lysed and subjected to luciferase assays to measure TOPFlash activity. Vec, pCAGGS plasmid vector; WT, pCAGGS plasmid encoding SFTSV wild type L protein. Comparison of mean values (A, D, and E) between two groups was analyzed by one-way ANOVA or two-way ANOVA analyses. (A–E) All experiments were performed with three independent replicates and any independent replicates had at least two technical replicates. Immunoblots (B and D) were representative of three independent experiments. The corresponding detected antibody of the band was labeled on the left and the marker size near the band was labeled on the lower right.

It has been reported that SFTSV L protein could be divided into three functional domains: the N-terminal Endo region, the middle RdRp core region, and the C-terminal CapB region (37, 38). To explore which functional domains account for the L protein-mediated inhibition of the WNT-CTNNB1 signaling pathway, point mutations were selected. D112, D1126/1127, and Q1707 have been reported to play vital roles in the Endo, RdRp, and CapB domains, respectively (38). As shown in Fig. 3A, the D112A mutant completely lost its inhibitory effect on the WNT-CTNNB1 signaling pathway, and the Q1707A mutant partially lost its inhibitory effect, while the inhibitory effect of the D1126/1127A mutant was basically the same as that of the wild type (Fig. 3A and B), suggesting that both Endo and CapB domains account for the downregulation of the WNT-CTNNB1 signaling pathway.

We then explored whether the inhibitor of SFTSV L protein CapB could affect the WNT-CTNNB1 signaling pathway. Based on the structural foundation of PB2 CapB inhibitor (VX-787) in influenza virus and CapB in SFTSV (39), we optimized and obtained a series of derivatives , and finally screened out one with good antiviral effects, Compound_34. Compound_34 could inhibit SFTSV replication *in vitro* in a dose-dependent manner (Fig. 3C and D). To further probe the binding mode between SFTSV CapB and Compound_34, we used Surfelx-dock to dock the small molecule into the m7GTP binding site (F1703, Q1707, Y1719, and L1772) (38). Through the examination of the best poses, it was suggested that Compound_34 could bind in the cap-binding pocket of SFTSV CapB (PDB:6XYA) and form a hydrogen bond interaction with Q1707 (Fig. S5). We found that Compound_34 could effectively alleviate the inhibitory effect of L protein on the WNT-CTNNB1 signaling pathway, while the RdRp inhibitor favipiravir (T-705) had no such effect (Fig. 3E).

Overall, point mutation targeting L protein Endo activity caused L protein to lose its inhibitory effect on the WNT-CTNNB1 signaling pathway, and CapB inhibitor could effectively alleviate this inhibitory effect. These complementary data showed that the SFTSV cap-snatching process would play an important role in inhibiting the WNT-CTNNB1 signaling pathway.

### SFTSV L protein reduces the intracellular protein level of CTNNB1

**Fig 4:**
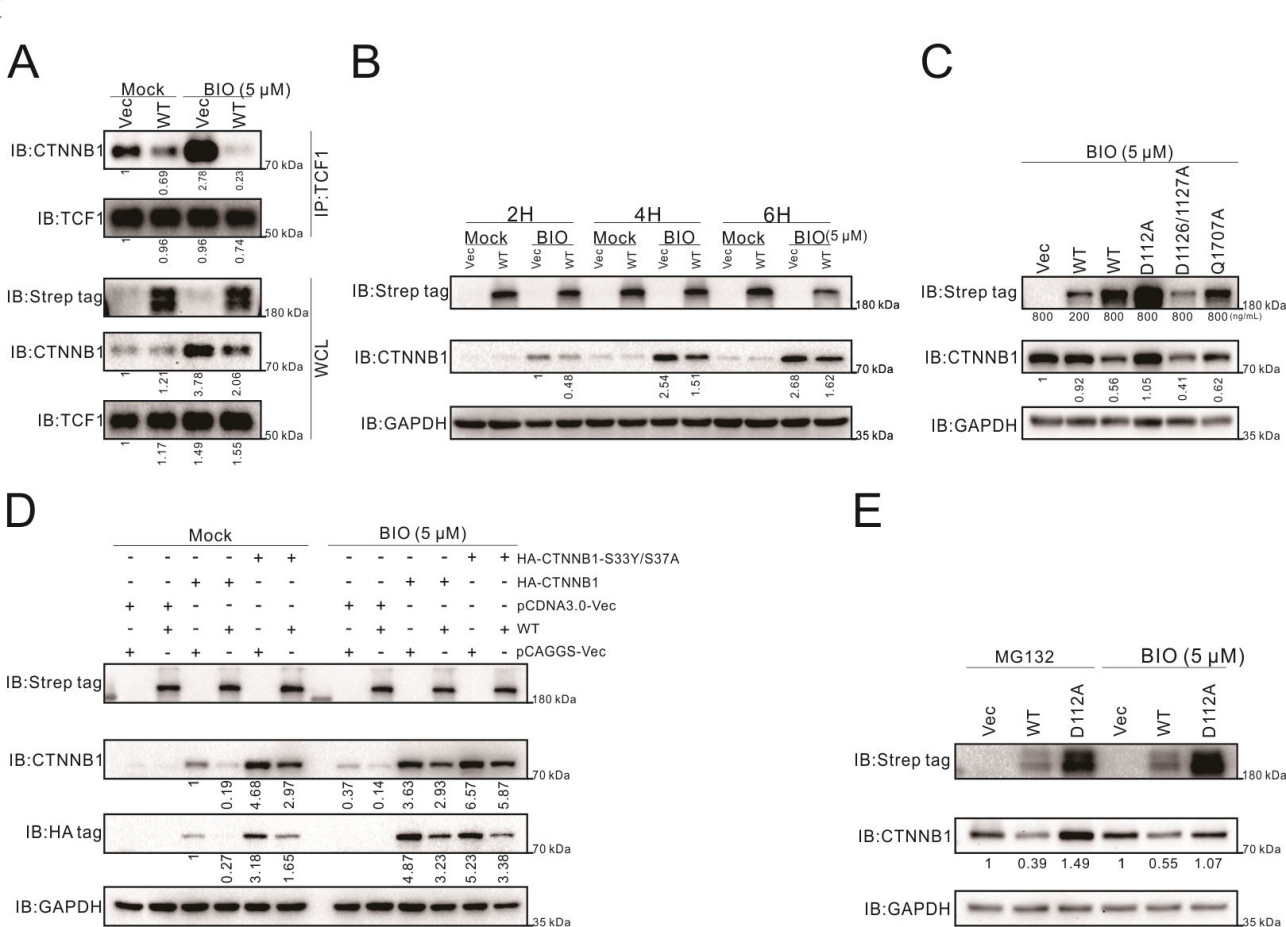
SFTSV L protein inhibits the WNT-CTNNB1 signaling pathway by reducing the intracellular protein level of CTNNB1. (A and B) SFTSV L protein reduced the intracellular protein level of CTNNB1. (A) Co-IP analysis of the interaction between CTNNB1 and TCF1. In six-well plates, HEK 293T cells were transfected with pCAGGS plasmids encoding SFTSV L protein or vector (1,600 ng/well). After 24-h post-transfection, cells were treated with BIO (5 µM). After 6 h, cells were harvested and lysed at 4°C. TCF1 was pulled down from the cell lysates by an anti-TCF1 antibody incubated with magnetic protein A/G beads. The precipitates were analyzed by Western blot analysis with antibodies specific to CTNNB1 and TCF1. SFTSV L proteins were detected by an anti-Strep tag antibody. WCL, whole cell lysate. (B) Time course assay of HEK 293T cells transfected with pCAGGS plasmids encoding SFTSV L protein or vector. At 24-h post-transfection, cells were nonactivated (mock) or activated (BIO, 5 µM) and as a time base, cells were collected for Western blot analysis at the indicated times (2 h, 4 h, and 6 h). SFTSV L protein and CTNNB1 were detected. GAPDH was also detected as a control for each lane. (C) SFTSV L protein decreased the overall level of CTNNB1 in a manner dependent on its cap-snatching activity. In six-well plates, HEK 293T cells were transfected with pCAGGS plasmids encoding SFTSV L proteins (wild type or mutants) or vector at the indicated concentrations, and supplemented with vector. At 24-h post-transfection, cells were treated (BIO, 5 µM). After 24 h, cells were collected for Western blot analysis. SFTSV L protein and CTNNB1 were detected. GAPDH was also detected as a control for each lane. (D and E) The intracellular protein level of CTNNB1 decreased by SFTSV L protein was independent of the degradation complex system and proteasome. (D) In six-well plates, HEK 293T cells were transfected with pCAGGS plasmids coding SFTSV L protein or vector (1,600 ng/well), and pCDNA3.0 plasmids coding human CTNNB1 proteins (wild type or mutant) with an N-terminal HA-tag or vector as a control (1,000 ng/well). At 24-h post-transfection, cells were untreated (mock) or treated (BIO, 5 µM). After 24 h, cell lysates were subjected to Western blot analysis with the indicated antibodies. GAPDH was also detected as a control for each lane. (E) In six-well plates, HEK 293T cells were transfected with pCAGGS plasmids coding SFTSV L proteins (wild type or D112A mutant) or vector (1600 ng/mL). At 24-h post-transfection, cells were treated with BIO (5 µM) or MG132 (10 µM). After 6 h, cell lysates were subjected to Western blot analysis with the indicated antibodies. GAPDH was also detected as a control for each lane. Immunoblots were representative of two or three independent experiments. The corresponding detected antibody of the band was labeled on the left, the marker size near the band was labeled on the lower right, and the transfection amount of the plasmid was also labeled below the band. Gray value analysis of the above bands using Image Lab. Relative quantitative results are shown below each corresponding band.

The central event of the WNT-CTNNB1 signaling pathway is the status of the degradation complex. CTNNB1 accumulates while the pathway is “ON”, and the interaction between CTNNB1 and TCF1 is a necessary process to drive downstream gene transcription (40). Therefore, to explore the effect of L protein on the WNT-CTNNB1 signaling pathway, first, the interaction between CTNNB1 and TCF1 in HEK 293T cells was detected by coimmunoprecipitation (Co-IP) assay, and it was found that the expression of L protein inhibited the interaction between them (Fig. 4A). We found that the overall level of CTNNB1 increased as a result of BIO activity, while SFTSV L protein alleviated this increase, not other viral proteins (Fig. 4B; Fig. S6). SFTSV L protein wild type and mutants of each functional domain were transfected into HEK 293T cells, corresponding to the previous results of the TOPFlash reporter system, the mutant D112A could not reduce CTNNB1 levels and Q1707A partially rescued CTNNB1 expression, while D1126/1127A reduced the level of CTNNB1 as well as the wild type (Fig. 4C).

The degradation complex consists of Axin, the adenomatous polyposis coli tumor suppressor protein, glycogen synthase kinase 3 (GSK3), and casein kinase 1. CTNNB1 is marked for degradation by GSK3-dependent phosphorylation at key amino-terminal Ser and Thr residues, including critical Ser33 and Ser37 (41). Phosphorylated CTNNB1 is then targeted for ubiquitination and proteasomal degradation (42). To further explore how L protein reduced the level of CTNNB1, HEK 293T cells were transfected with wild-type CTNNB1 or the S33Y/S37A mutant, which cannot be degraded by the proteasome, and we found that L protein could reduce the exogenous expression levels of both wild-type CTNNB1 and the S33Y/S37A mutant, detected by HA-tag (Fig. 4D). Reported studies have shown that the degradation of CTNNB1 depends on the proteasome system. We found that treatment with MG132, a proteasome inhibitor, resulted in the accumulation of intracellular CTNNB1 but did not alleviate the reduction in its level due to L protein (Fig. 4E) or other major protein degradation pathways (Fig. S7). Taken together, SFTSV L protein inhibited the WNT-CTNNB1 signaling pathway by downregulating the intracellular protein level of CTNNB1, and this process might be independent of the degradation complex and proteasome system.

### SFTSV L protein interacts with mRNAs of WNT-CTNNB1 signaling pathway-related genes

**Fig 5:**
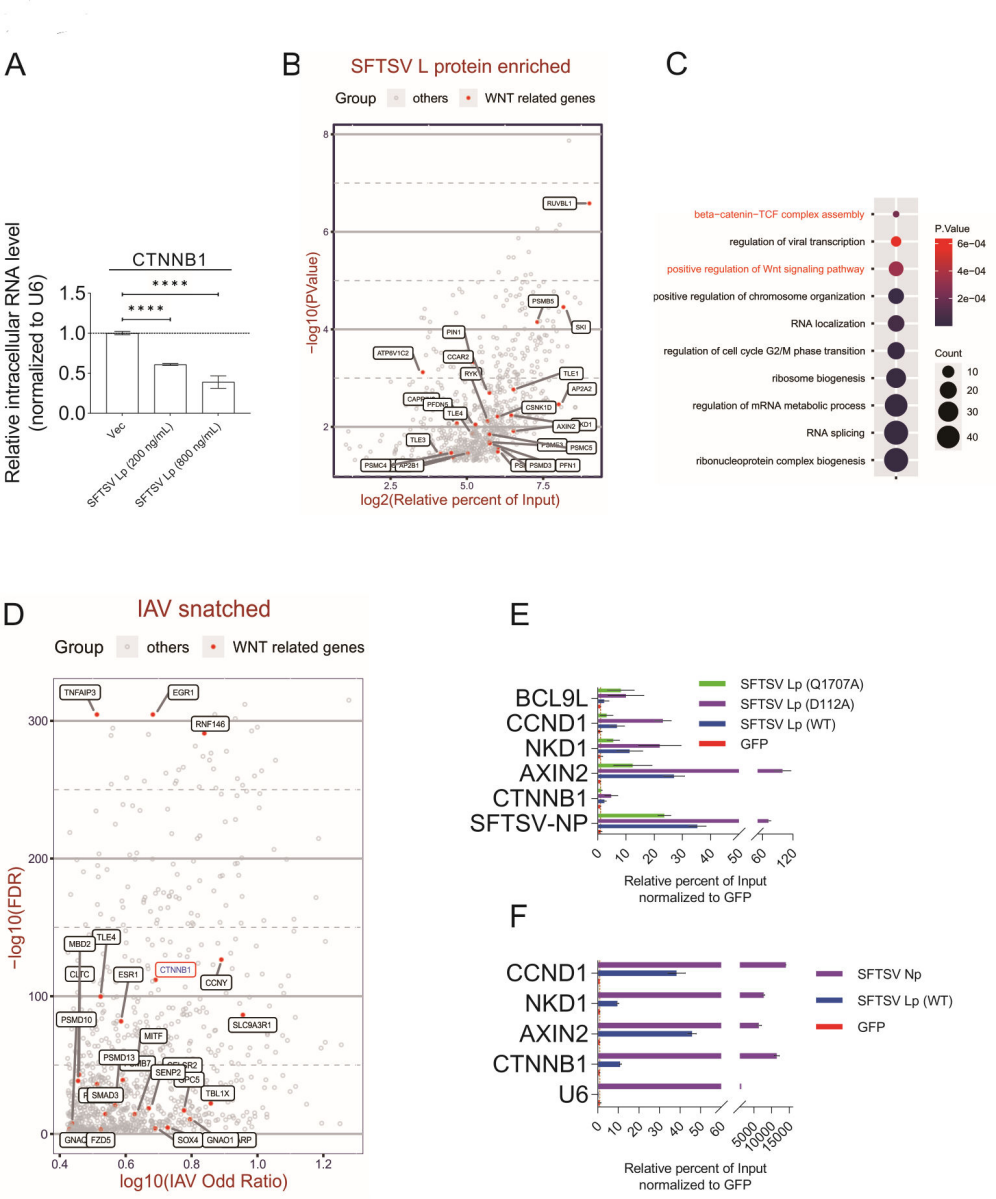
SFTSV L protein interacts with mRNAs of *CTNNB1* and other WNT-CTNNB1 signaling pathway-related genes. (A) SFTSV L protein downregulated *CTNNB1* mRNA in HEK 293T cells. In six-well plates, HEK 293T cells were transfected with pCAGGS plasmids encoding SFTSV L protein. At 24-h post-transfection, cells were treated with BIO (5 µM). After 6 h, cells were collected and intracellular RNA was extracted. Quantitative real-time PCR (qRT-PCR) analysis of *CTNNB1* mRNA normalized to *U6*. (B and C) Plot and GO analysis of mRNAs enriched in SFTV L protein. In 10-cm dishes, HEK 293T cells were transfected with pCAGGS plasmids encoding SFTSV L protein or Green fluorescent protein (GFP) with C-terminal Twin-Strep-tag (10 µg/dish). At 24-h post-transfection, cells were activated (BIO, 5 µM) and infected with SFTSV (MOI = 1) for another 6 h. Then, cells were subjected to RNA immunoprecipitation (RIP) with MagStrep XT beads, followed by RNA extraction and sequencing. (B) Red and gray indicate SFTSV L protein-enriched RNAs of WNT-related genes and other genes [log2 (relative percentage of input) ≥ 1, *P* < 0.05], respectively. The WNT-related genes were mapped, as shown. The test was performed using the Fisher’s exact test. Relative percentage of input, the relative percentage of SFTSV L protein input (input counts/output counts) normalized to the percentage of GFP input (input counts/output counts). (C) Representative overview of GO analysis based on biological process annotations. Subsets of SFTSV L protein-enriched genes were screened for GO analysis. WNT-related GO terms of interest were highlighted in red. (D) Red and gray indicate IAV snatched RNAs of WNT-related genes and other genes, respectively. The WNT-CTNNB1 signaling pathway-related genes were indicated and the “CTNNB1” was blue and circled in red, as shown. IAV Odd Ratio: odds ratio of IAV snatched versus IAV unsnatched. The test was performed using Fisher’s exact test. (E and F) RIP/qRT-PCR analysis of SFTSV L protein (wild type or mutants) interacting mRNAs. (E) In 10-cm dishes, HEK 293T cells were transfected with pCAGGS plasmids coding SFTSV L proteins (wild type or mutants) or GFP with C-terminal Twin-Strep-tag (10 µg/dish). At 24-h post-transfection, cells were activated (BIO, 5 µM) and infected with SFTSV (MOI = 1) for 6 h. Then, the cells were subjected to RIP with MagStrep XT beads, followed by immunoblotting and RNA extraction. RNA extracted from RNA immunoprecipitation was used to measure the transcription of the indicated genes with quantitative real-time PCR (qRT-PCR). The SFTSV L protein relative percentage of input was calculated based on the percentage of GFP input. (F) In 10-cm dishes, HEK 293T cells were transfected with pCAGGS plasmids coding SFTSV L protein, N protein, or GFP with C-terminal Twin-Strep-tag (10 µg/dish). At 24-h post-transfection, cells were activated (BIO, 5 µM) for 6 h. Then, the cells were subjected to RNA immunoprecipitation (RIP) with MagStrep XT beads, followed by immunoblotting and RNA extraction. RNA extracted from RNA immunoprecipitation was used to measure the transcription of the indicated genes with quantitative real-time PCR (qRT-PCR). The SFTSV L or N protein relative percentage of input was calculated based on the percentage of GFP input. GFP, pCAGGS plasmid encoding GFP; Lp or Np, pCAGGS plasmid encoding SFTSV L or N protein. Comparison of mean values (A) between two groups was analyzed by one-way ANOVA analyses. (A, E, and F) All experiments were performed with three independent replicates and any independent replicates had at least two technical replicates. And the results (E and F) were representative of three independent experiments.

According to the above results, it can be speculated that the decreased protein level of CTNNB1 might be due to its decreased mRNA level. Our quantitative real-time PCR (qRT-PCR) analysis indicated that *CTNNB1* mRNA levels were downregulated in HEK 293T cells expressing SFTSV L protein (Fig. 5A). RNA immunoprecipitation (RIP) combined with high-throughput sequencing or qRT-PCR can sensitively detect RNAs bound to the target protein (43). Thus, we used RIP sequencing analysis to detect SFTSV L protein-interacting RNAs and found that the RNAs of some WNT-related genes were enriched (Fig. 5B). The WNT-related genes were highlighted in the plot, and more L protein interacting genes were described in Table S1. GO analysis was conducted on the L protein-enriched genes (Table S2), among which the WNT-related categories were overrepresented, “beta-catenin-TCF complex assembly” (*P* = 0.0002) and “positive regulation of Wnt signaling pathway” (*P* = 0.0003) (Fig. 5C). Also, further analysis of top 1,000 genes snatched in a reported IAV cap analysis of gene expression (CAGE)-sequencing data set (44) showed that some RNAs related to the WNT-CTNNB1 signaling pathway could be snatched by IAV (Fig. 5D). Meanwhile, SFTSV L protein and its mutants showed differences in RNA enrichment (Fig. 5E; Fig. S8A), among which Q1707A but not D112A could affect its binding to multiple mRNAs, suggesting that L protein-mediated regulation of WNT-CTNNB1 signaling pathway was attributed to both CapB and Endo. And SFTSV L protein could interact with the mRNAs of WNT-CTNNB1 signaling pathway-related genes independently of its N protein (Fig. 5F; Fig. S8B). Altogether, our results indicated that L protein might interact with mRNAs of WNT-CTNNB1 signaling pathway-related genes by its CapB, and its Endo activity might decrease the mRNA level of *CTNNB1*.

### The WNT-CTNNB1 signaling pathway can affect SFTSV replication *in vitro* and *in vivo*

**Fig 6:**
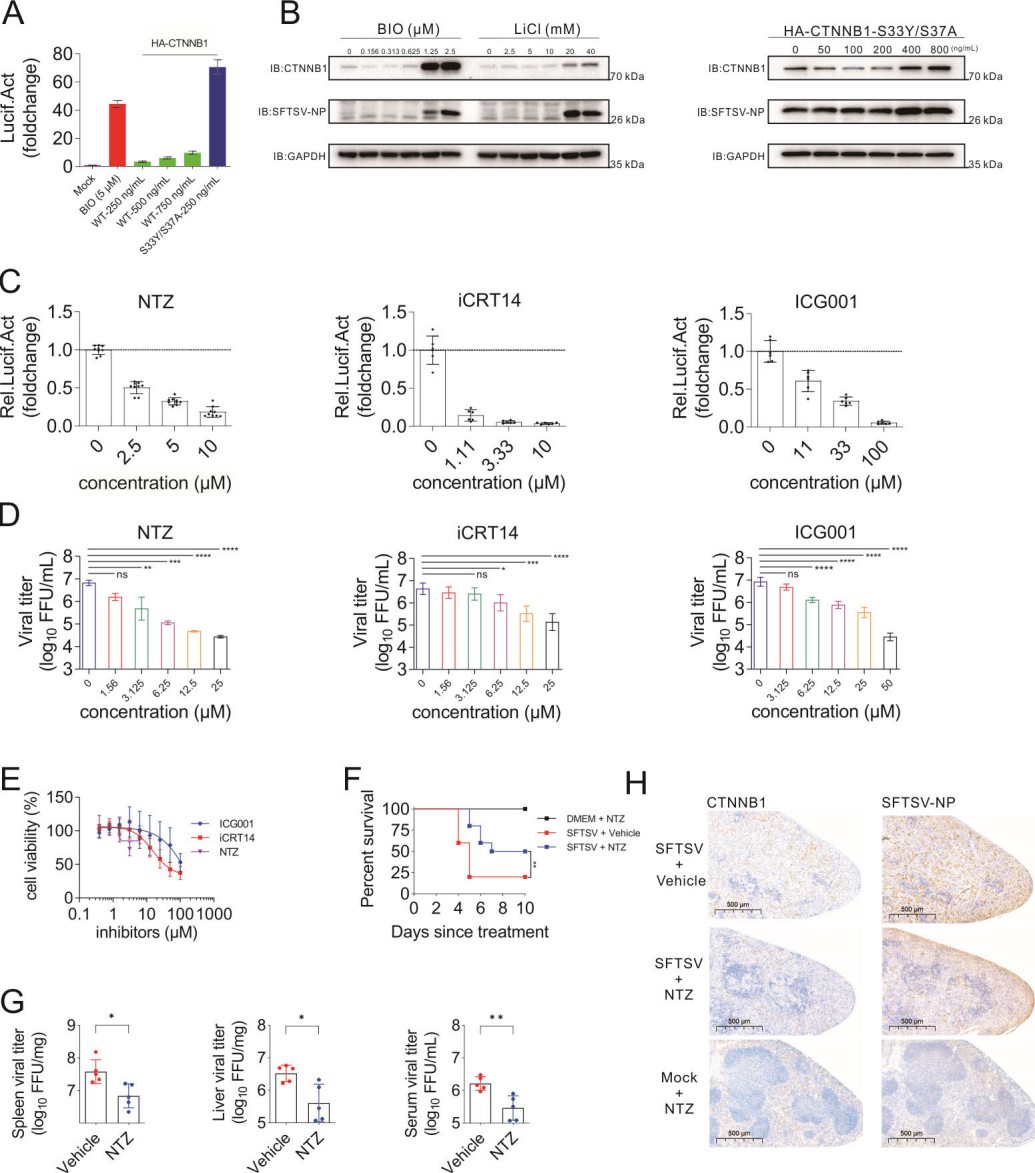
Modulation of the WNT-CTNNB1 signaling pathway affects SFTSV replication *in vitro* and *in vivo*. (A and B) Activation of the pathway promoted SFTSV replication in HEK 293T cells. (A) In 48-well plates, HEK 293T cells were transfected with TOPFlash reporter plasmids (50 ng/well), pRL-TK internal control luciferase reporter plasmids (50 ng/well), and pCDNA3.0 plasmids coding CTNNB1 (wild type or S33Y/S37A) or vector at the indicated amounts, and supplemented with vector. At 24-h post-transfection, cells transfected with vector were treated (BIO, 2.5 µM) as a positive control. After 24 h, the cells were lysed and subjected to luciferase assays. (B) In 24-well plates, HEK 293T cells overexpressing the indicated gradient amounts of pCDNA3.0 plasmids coding CTNNB1 (S33Y/S37A) for 24 h were infected with SFTSV at an MOI of 1. After another 36 h, the cells were collected and subjected to Western blot analysis (left). In 24-well plates, HEK 293T cells were treated with a series of concentrations of BIO or LiCl for 24 h. After another 36 h, the cells were collected and subjected to Western blot analysis with the indicated antibodies (right). Tubulin was also detected as a control for each lane. (C–E), WNT-CTNNB1 signaling pathway inhibitors inhibited SFTSV replication. (C) TOPFlash assay of HEK 293T cells treated with iCRT14, ICG001, or NTZ. In 48-well plates, HEK 293T cells were transfected with TOPFlash reporter plasmids (50 ng/well) and pRL-TK internal control luciferase reporter plasmids (50 ng/well). At 24-h post-transfection, cells were treated with iCRT14, ICG001, or NTZ at the indicated concentrations. After 48 h, the cells were lysed and subjected to luciferase assays. (D) Evaluation of the effect of the compounds on SFTSV replication. In 24-well plates, HEK 293T cells were pre-infected with SFTSV at an MOI of 0.1 for 1 h. The cells were treated with a series of concentrations of these compounds. After 48 h, supernatants of cells treated with each concentration of these compounds were collected for viral titer determination. Detailed methods and procedures for viral FFU determination are in the Materials and Methods. (E) The cytotoxicity of these compounds in HEK 293T cells was also determined by CCK8 assay. The four-parameter dose–response curve was fitted using the nonlinear regression method in the software Prism 9.0. (F–H) NTZ inhibited SFTSV replication by decreasing CTNNB1 *in vivo*. (F) The effect of NTZ on SFTSV-induced fatality in mice. Kaplan–Meier curves of the NTZ treatment effect on the probability of survival in C57BL/6 mice. C57BL mice were divided into three groups: SFTSV + vehicle group (10), SFTSV + NTZ group (10), and DMEM (Dulbecco’s modified Eagle’s medium) + NTZ group (5). (G) Serum, spleen, and liver samples were collected, and a titration of virus was determined by immunostaining plaque assay. (H) Representative immunohistochemistry (IHC) staining of CTNNB1 and SFTSV NP in spleen samples. Comparison of mean values (D and G) between two groups was analyzed by unpaired *t*-test or one-way ANOVA analyses. (A–E) All experiments were performed with three independent replicates and any independent replicates had at least two technical replicates. The immunoblots (B) were representative of three independent experiments. The corresponding detected antibody of the band was labeled on the left and the marker size near the band was labeled on the lower right.

We then explored whether modulation of the WNT-CTNNB1 signaling pathway can affect SFTSV replication. First, we found that overexpression of CTNNB1 could activate the WNT-CTNNB1 signaling pathway (Fig. 6A). In particular, S33Y/S37A mutant CTNNB1 and overexpression of S33Y/S37A mutant CTNNB1 or treatment with BIO or LiCl could promote viral replication (Fig. 6B). In addition, there are some reported inhibitors of the WNT-CTNNB1 signaling pathway. iCRT14 can appropriately reduce DVL2 without affecting its phosphorylation, and also affect TCF-CTNNB1 interactions (45); ICG-001 antagonizes WNT/CTNNB1/TCF-mediated transcription and specifically binds to promoter binding protein (46); nitazoxanide (NTZ) can increase the citrullination and turnover of CTNNB1 in cancer cells (47). They could effectively inhibit TOPFlash activity at the indicated concentrations (Fig. 6C) and SFTSV replication in a dose-dependent manner (Fig. 6D and E; Fig. S9). NTZ could also effectively inhibit IAV replication in Madin Darby Canine Kidney (MDCK) cells (Fig. S10). Simultaneously, knockdown of DVL2, a positive regulator of the WNT-CTNNB1 signaling pathway (22), could effectively inhibit the activation of the WNT-CTNNB1 signaling pathway, thereby reducing SFTSV replication (Fig. S12). We further found that RNAi-mediated knockdown or CRISPR/Cas9-mediated knockout of CTNNB1 could effectively inhibit SFTSV replication (Fig. S13). In conclusion, the WNT-CTNNB1 signaling pathway is essential for SFTSV replication *in vitro*.

Furthermore, NTZ treatment effect on SFTSV infection was chosen to analyze fatal outcomes in an SFTSV lethal SFTSV mouse model. C57BL/6 mice were pre-treated with anti-IFNAR1 (interferon alpha/beta receptor 1) immunoglobulin G (IgG) and then challenged intraperitoneally with SFTSV. At 1 h post-infection, mice were intragastrically administered NTZ (*n* = 10) or an equal volume of vehicle (*n* = 10). Mice treated with NTZ only (*n* = 5) were included as the negative control. The survival rates of mice were significantly increased after treatment with NTZ compared to those treated with vehicle (50% versus 20%, *P* < 0.01, log-rank test; Fig. 6F). Significantly reduced viral titers of SFTSV, measured by focus forming assay on Vero cells, were observed in the serum, spleen, and liver samples of NTZ-treated mice (Fig. 6G). Notably, immunohistochemistry (IHC) analysis revealed decreased protein levels of both CTNNB1 and SFTSV NP in spleen tissue (Fig. 6H; Fig. S11). These results suggest that an inhibitor of the WNT-CTNNB1 signaling pathway can inhibit SFTSV replication *in vivo*.

## DISCUSSION

Replication and transcription of sNSVs, including *de novo* synthesis replication and “cap-snatching” initiation transcription, are carried out in the ribonucleoprotein (48). The m7Gppp cap at the 5′-end can stabilize host mRNA, while sNSVs can snatch the host mRNA cap as a primer for transcription initiation (49), and mRNA will be degraded immediately after the loss of caps (50–52). Currently, studies have reported that influenza virus prefers host small nuclear RNAs (44), and certain bunyaviruses prefer mRNAs of host cell cycle-related genes (53, 54). The WNT-CTNNB1 signaling pathway plays an important role in cell proliferation and differentiation (55, 56). Many genes in the pathway are related to the cell cycle (57) and antiviral immunity (58–60). Under normal steady-state conditions, the WNT-CTNNB1 signaling pathway is in a silent state. Simultaneously, the WNT-CTNNB1 signaling pathway has a feedback regulation mechanism, which maintains the activation degree of the pathway and its related physiological activities within the acceptable range (61, 62).

In this study, we performed transcriptomic analysis of blood samples collected from SFTS patients and found that the WNT-CTNNB1 signaling pathway was significantly downregulated at the acute phase of infection in patients who eventually succumbed to SFTSV infection. Considering that the pathway and its downstream transcription factors play important roles in the proliferation and differentiation of T-cells (63, 64), and severe disease is associated with T-cell dysregulation that may hinder the mounting of an immune response and subsequent clearance of the viral infection (65), it was inferred that SFTSV may affect the differentiation, composition, and function of T-cells in SFTS patients, which may lead to the exacerbation of disease and even death of patients. Subsequent studies found that both Endo and CapB domains of L protein account for the downregulation of WNT-CTNNB1 signaling pathway. D112A mutation, which is vital for Endo, could rescue the activation of WNT-CTNNB1 signaling pathway. In addition, we tried to use RIP-seq to find out the target genes that might be used as substrates for viral cap-snatching by obtaining host RNAs interacting with SFTSV L protein (43). By comparing our results with those of IAV CAGE-seq data (44), we did not find many genes in common. This is due to the fact that RIP-seq focuses more on interacted mRNA, while CAGE-seq prioritizes chimera mRNA. However, it should be noted that these results are not directly comparable but complementary, as the cellular localization of L protein or polymerase complex is also different between SFTSV and IAV. Nevertheless, related information suggests that utilizing a combination of multiple sequencing methods may be a promising approach to identify cap-snatched genes of sNSVs. Together, these results indicated that SFTSV L protein could interact with multiple WNT-CTNNB1 pathway-related RNAs and reduce the mRNA level of *CTNNB1*, resulting in a decrease in the overall CTNNB1 protein level. In summary, SFTSV could specifically regulate the WNT-CTNNB1 signaling pathway in a cap-snatching manner, and this pathway plays an important role in viral replication, or mRNAs related to the pathway might be targets of viral cap-snatching. Similar to SFTSV, we found that LASV L protein and IAV polymerase complex could also inhibit the WNT-CTNNB1 signaling pathway (Fig. 2F), although at different levels, and NTZ could inhibit IAV replication (Fig. S10). It was hypothesized that the regulation of the WNT-CTNNB1 signaling pathway in a cap-snatching manner observed in the life cycle of SFTSV might be conserved in other sNSVs (15, 17, 53–55). As for potential pathogenesis, it is worth noting that the WNT-CTNNB1 signaling pathway also plays a role in regulating various aspects of platelet function, including adhesion, activation, dense granule secretion, aggregation, and formation, as indicated by studies (66, 67). Indeed, thrombocytopenia is a primary and typical feature of SFTSV infection in human beings and is related to the fatal outcomes of patients with SFTS. These findings naturally raise questions about whether the virus’s manipulation of this pathway could influence the development of pathological conditions. Therefore, additional clinical data from patients with SFTS are essential for a deeper understanding of the relationship between the WNT-CTNNB1 pathway and SFTSV-induced thrombocytopenia. And exploring the use of cap-snatching inhibitors, like Compound_34 or the pathway inhibitors, as potential treatments for viral infections might be a promising avenue for future trials.

**Fig 7:**
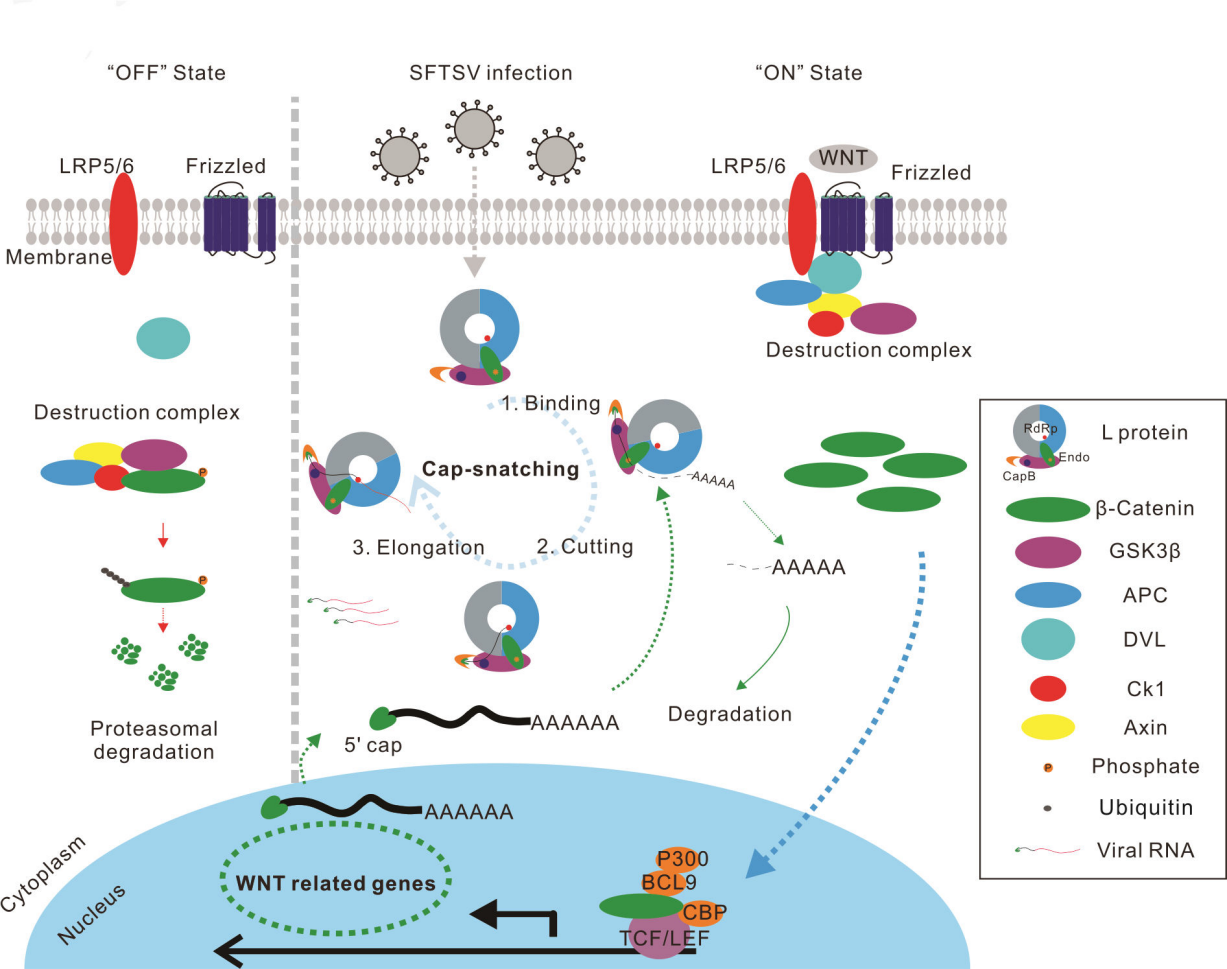
Schematic model illustrating regulation of the WNT-CTNNB1 signaling pathway by SFTSV in a cap-snatching manner. Schematic demonstrating multiple potential mechanisms that genes related to the WNT-CTNNB1 signaling pathway could be utilized as a primer “pool” for SFTSV cap-snatching (17) and SFTSV could regulate the state of the pathway in a cap-snatching manner. SFTSV performs cap-snatching in the cytoplasm and viral L protein is responsible for cap binding and cleavage of host mRNA 10–14 bp nucleotides downstream of the 5’-cap (68). This short, capped RNA fragment is subsequently used as a primer for viral transcription. This results in a chimeric mRNA, which is then translated into viral proteins (76). As a result, the host mRNAs whose cap is snatched by SFTS L protein lose the protection of the 5’-cap and will be degraded (50, 52, 77).

CapB and Endo of L protein play key roles in cap-snatching process. In this process, CapB captures the host mRNA and Endo is capable of cleaving short 5′-capped RNA fragments of host cell mRNAs, which are then used to prime the synthesis of viral mRNA sequences in the RdRp catalytic cavity (Fig. 7). The active region of Endo domain between sNSVs is highly conserved with a motif (PD…D/E…K) (68). We aligned the CapB domain (CBD) sequences of sNSVs from multiple families (data not shown), showing low similarity between sequences except for some conserved sites, although the key residues responsible for interaction with m7GTP were functionally conserved between phenuiviruses (38). Here, we found Q1707A and the CapB inhibitor could partially or effectively rescue WNT-CTNNB1 signaling pathway activity, and it has been reported that F1703, Y1719, and Q1707 play important roles in binding m7GTP to SFTSV CapB domain, suggesting that single point mutation may not lead to loss of CapB function completely and mutations introducing multiple sites need to be considered. The structural distinctions between viral CapB and Endo suggest variations in the cap-snatching process among different sNSVs. These distinctions also account for the differences in how SFTSV, IAV, and LASV L-protein or polymerase complexes inhibit TOPFlash activity (Fig. 2F). Firstly, there is a significant difference in the affinity between CapB and m7GTP, which would result in competition with other cap-binding proteins within the host (69). Secondly, the presence of histidine at the active sites of the two types of endonucleases, namely His+ and His−, plays a pivotal role in the endonuclease activity. LASV endonuclease belongs to the latter category, providing a plausible explanation for the variations in the effects of the L protein on the host and the virulence determined by the L RNA segment (70–72). All these features provide the structural basis for the broad-spectrum mechanism of the regulation of the WNT-CTNNB1 signaling pathway in a cap-snatching manner among sNSVs. In addition, the differences in cap-snatching process are also related to the viral replication cycle (49, 73, 74). Presumably, the interaction between the pathway and sNSVs could bridge these differences. Recent study has shown that MTr1, which is used for RNA cap modification in the host, was essential for IAV replication, playing an important role in cap-snatching for IAV. The screened inhibitor TFMT could effectively inhibit IAV replication (75), which provides the possibility for the development of novel antiviral drugs based on correlation between host and the cap-snatching of sNSVs. Given the importance of the cap-snatching process for sNSVs transcription and replication, as well as its host dependence, further study of this process is extremely important for the development of effective and broad-spectrum antiviral drugs.

## MATERIALS AND METHODS

### Transcriptomic analyses of blood samples from SFTS patients

To investigate the pathogenesis mechanisms of SFTSV infection, we performed transcriptomic analysis with blood samples collected from laboratory-confirmed SFTS patients. For the included survival patients (*n* = 23), deceased patients (*n* = 17), and healthy controls (*n* = 23), the mean (standard deviation, SD) age was 64.6 ± 8.7, 66.7 ± 8.4, and 60.1 ± 7.3 years, respectively, and the proportion of female was 70%, 59%, and 57%, respectively. The median (interquartile range, IQR) from symptom onset to admission was 6 (4–6) and 6 (5–7), and the median (IQR) viral load (log10 copies/mL) was 6.97 (6.53–7.87) and 7.56 (6.64–9.01), for the survival and deceased patients, respectively. The median time from symptom onset to sampling was 15 (13–16) for the recovered patients. The recruited patients had their peripheral blood samples collected using the PAXgene Blood RNA tube (BD Biosciences) at the acute phase of illness when admitted to the hospital and at the convalescent phase when discharged from the hospital.

RNA-seq data sets are available at the NCBI under the accession number GEO: GSE144358 (27). The raw count matrix from RNA-Seq data sets was filtered for low expressed genes (total less than 10 counts). *t*-SNE analysis was performed using the Rtsne R package version 0.16. Differential gene expression analysis was performed using the DESeq2 R package version 1.40.0 (78). The parameters for classifying significant DEGs were twofold differences (|log_2_FC| ≥ 1, FC: the fold change of expression) in transcript abundance and *P*-value <0.05. Subset of genes screened for gene ontology (GO) analysis was performed using the clusterProfiler R package version 4.0 (79). Visualization of analysis results and other analysis of data were performed using R 4.0.0.

### Cell culture and virus infection

HEK 293T cells (ATCC), MDCK cells (ATCC), and Vero cells (ATCC) were cultured in Dulbecco’s modified Eagle’s medium (DMEM; Thermo Fisher Scientific, Waltham, MA, USA). Cells were routinely cultured at 37°C with 5% CO_2_, and all media were supplemented with 10% (vol/vol) fetal bovine serum (Gibco). SFTSV strain HNXY2017-66 was stored in our laboratory.

Cells were inoculated with SFTSV at the indicated MOI for virus infection. After adsorption for 1.5 h at 37°C, the cells were washed with phosphate-buffered saline (PBS) and cultured in DMEM containing 2% serum.

### Evaluation of the cytotoxicity of the test compounds

In 96-well plates, HEK 293T cells were treated with the different concentration of the indicated compounds, and 48 h later, the relative numbers of surviving cells were measured with cell counting kit-8 (GK10001, GLPBIO) according to the manufacturer’s instructions.

### Immunological focus assay

Viral titer was determined by focus forming assay on Vero cells. Briefly, confluent monolayers were incubated with 10-fold dilutions of virus for 1.5 h, and then the culture medium was replaced by DMEM containing 2% serum and supplemented with 1.25% carboxymethyl-cellulose. At 4 days post-infection, the cells were fixed with 4% paraformaldehyde for 30 min at room temperature, permeabilized, and blocked in 5% defatted milk containing 0.3% Triton X-100 (dissolved in PBS) for 1 h at room temperature. Next, the cells were incubated with anti-NP serum (1:2,000 dilution) for 1 h at room temperature or 4°C overnight. Then, the plates were washed extensively in wash buffer (PBS containing 0.05% Tween 20) three times for 5 min each, followed by incubation with an HRP (horseradish peroxidase)-conjugated anti-rabbit secondary antibody (Proteintech, SA00001-2, 1:1,000 dilution) for 1 h at room temperature. Finally, the cells were washed three more times, and viral focus forming units were stained using an enhanced HRP-DAB (3,3’ Diaminobenzidine Tetrahydrochloride) chromogenic substrate kit (Tiangen Biotech, PA110). Viral titers were determined based on the number of focus-forming units (FFU) (80).

### Reagents and antibodies

The activators LiCl (Sinopharm Chemical Reagent, 7447-41-8), BIO (Selleck Chemical, S7189), inhibitors iCRT14, ICG001, Z-VAD-FMK, chloroquine (Selleck Chemical, S8704, S2662, S7023, S6999) and NTZ (MedChemExpress, HY-B0217), and proteasome inhibitor MG132 (MedChemExpress, HY-13259) were used. The Dual-Luciferase Reporter Assay System (Promega, E2940), protease inhibitor cocktail (Beyotime, P1005), MagStrep XT beads (IBA Lifesciences, 2-4090-002), and Protein G magnetic beads (Bio-Rad, 161-4032) were used. Rabbit anti-strep tag pAb (Genescript, A00626, 1:1,000) and rabbit anti-HA tag pAb (Proteintech, 51064-2-AP, 1:2,000); rabbit anti-CTNNB1 mAb (Abcam, SP328, 1:400), rabbit anti-TCF1 mAb (Cell Signaling Technology, 2203T, 1:1,000), rabbit anti-DVL2 pAb (Proteintech, 12037-1-AP, 1:1,000), rabbit anti-IAV Nucleoprotein mAb (GeneTex, GTX636318, 1:1,000); mouse anti-GAPDH mAb (Proteintech, 60004-1-Ig, 1:4,000), and rabbit anti-tubulin pAb (Proteintech, 14555-1-AP, 1:4000) were used. HRP-conjugated Affinipure goat anti-rabbit IgG (H + L) (Proteintech, SA00001-2, 1:10,000) and HRP-conjugated Affinipure goat anti-mouse IgG (H + L) (Proteintech, A00001-1, 1:10,000) were used. The antibodies were purchased from the indicated manufacturers. Rabbit polyclonal antibodies against SFTSV NP were raised against NP protein.

### Plasmids and transfection

Standard molecular biology procedures were performed for all plasmid constructions. The TOPFlash plasmid (TCF Reporter Plasmid, 21-170) was obtained from Sigma-Aldrich. Plasmids for viral protein expression, pCAGGS-SFTSV Lp/Gn/Gc/NSs/Np-TS, pCAGGS-LASV Lp-TS, pCAGGS-PR8 PA/PB1/PB2-TS, and pCAGGS-GFP-TS, pRL-TK internal control luciferase reporter plasmid, were previously generated by our lab. pCDNA3.0-HA-CTNNB1 (PVT10364) was purchased from the LifeScience market. The mutants were generated by PCR-directed site mutagenesis using KOD-Plus-Neo (KOD-201, TOROBO) and enzyme FastDigest DpnI digestion (FD1703, Thermo Fisher Scientific). Plasmids for the lentivirus package and CRISPR/Cas9-based gene knockout, including pMD2.G, pCMV-dR8.91, and lentiCRISPRv2 were gifts from Professor Ke Peng (Wuhan Institute of Virology, Chinese Academy of Science). sgRNA sequences specific to *CTNNB1* were cloned into lentiCRISPRv2 according to a standard target sequence cloning protocol described below (81). All of the plasmids were confirmed by Sanger sequencing. The amounts of plasmids used in each experiment are supplemented in figure legends.

### ELISA and immunofluorescence microscopy

Concentrations of secreted Wnt7a and Wnt10b in the serum samples were determined using the human protein Wnt-7a (WNT7A) ELISA kits (Fine Test, EH2312) and Wnt-10b (WNT10B) ELISA kits (Aviva Systems Biology, OKCD09426) following the manufacturer’s instructions.

To detect the intracellular expression level of viral NP, cells were fixed with 4% paraformaldehyde in advance. Fixed cells were permeabilized with 0.3% Triton X-100 and blocked with 5% bovine serum albumin. Then they were incubated for 2 h with the anti-IAV NP (1:1,000 dilution) as the primary antibody, followed by incubation with Alexa 488-labeled goat anti-rabbit IgG (Abcam, ab150077; 1:500 dilution). The nuclei were stained with DAPI (Sigma-Aldrich, D9542). The images were taken by fluorescence microscopy.

### Transfection and luciferase assay

According to the manufacturer’s recommendations for transient expression, HEK 293T cells were transfected with the indicated plasmids using Lipofectamine 2000 transfection reagent (Thermo Fisher Scientific). HEK 293T cells were plated in 48-well plates and transfected with a mixture of luciferase reporter (TCF reporter plasmid, 50 ng/well) and pRL-TK (Renilla luciferase plasmid, 50 ng/well) together with the indicated plasmids. At 24 h post-transfection, the cells were treated with activators, inhibitors, or other compounds for 24 h. The cells were lysed with Passive Lysis Buffer (Promega, E1941), and the luciferase was measured with a Dual-Glo Luciferase Assay System (Promega, E2940) according to the recommended protocol. The firefly luciferase reporter activity was normalized to the control Renilla activity.

### Immunoblotting and Co-IP

Samples were prepared and treated as described above, and cell monolayers were washed with PBS and lysed with lysis buffer containing 50 mM Tris-HCl (pH 7.5), 150 mM NaCl, 5 mM EDTA, 0.5% NP-40, and protease inhibitor cocktail (Beyotime, P1005). The protein concentrations were determined using a BCA analysis kit (Beyotime, P0012). Cell lysates were denatured by adding loading buffer (containing β-mercaptoethanol) followed by heating for 15 min. Protein fractions were loaded onto an SDS-PAGE gel to perform immunoblotting and transferred onto nitrocellulose filter membrane (Millipore, INYC00010) according to the manufacturer’s instructions. The membranes were blocked in 5% defatted milk (dissolved in Tris-buffered saline) for 1 h at room temperature and then incubated with a primary antibody for 1.5 h at room temperature or overnight at 4°C. The membranes were then washed extensively in wash buffer (TBS containing 0.05% Tween 20) three times (for 5 min each time) with agitation and incubated with an HRP-conjugated secondary antibody for 1 h at room temperature according to the species source of the primary antibody. The membranes were washed three times in wash buffer and imaged using an enhanced chemiluminescence substrate solution (Millipore, 34075) to visualize the protein bands.

For transient transfection and coimmunoprecipitation experiments, HEK 293T cells were transfected with the indicated plasmid for 24 h. The transfected cells were lysed in 0.5 mL of IP lysis buffer (Thermo Fisher Scientific, 87787). The supernatant was incubated with the indicated antibodies at 4°C for 4 h. Immune complexes were precipitated by incubating Protein G magnetic beads for 6 h at 4°C. After three stringent washes in PBS-T (0.05% Tween-20), immunoprecipitated proteins were analyzed by immunoblotting.

### RNA interference

The sequences of siRNAs targeting different regions of the mRNA transcripts were 5′-CUAGUCAACCUGUCUCUCATT-3′ (siDVL2#1), 5′-GAGACAGAAACCGAGUCAGTT-3′ (siDVL2#2), and 5′-GCAGCUGGAAUUCUUUCUATT-3′ (siCTNNB1). siRNA without human mRNA targets was used as a negative control (NC) for RNAi-related experiments. All siRNAs used in experiments were synthesized by genePharma (China). siRNA was transfected into HEK 293T cells using Lipofectamine RNAiMAX transfection reagent (Thermo Fisher Scientific, 13778500) with maintenance medium. At 48-h post-transfection, cells were infected with virus or re-transfected with the indicated plasmids. The amounts of siRNAs used in each experiment are supplemented in figure legends.

### RIP-seq

Two 10-cm plates of 95% confluent HEK 293T cells were used for each sample. Cells were washed three times with ice-cold PBS and scraped off the plates, followed by centrifugation at 800 × *g* for 3 min at 4°C. The cell pellets were resuspended in 1 mL of RIP buffer [150 mM KCl, 25 mM Tris-HCl pH 7.4, 5mM EDTA, 0.5 mM dithiothreitol (DTT), 0.5% NP-40, 100 U/mL RNase inhibitor, 100 µM PMSF, and protease inhibitor cocktail]. The lysates were centrifuged at 16,000 × *g* for 10 min, and the supernatant containing the protein‒RNA complexes was incubated with precleared MagStrep XT beads overnight at 4°C. The beads were then washed three times each with washing buffer (300 mM KCl, 25 mM Tris-HCl pH 7.4, 5 mM EDTA, 0.5 mM DTT, 0.5% NP40, 100 U/mL RNase inhibitor, 100 µM PMSF, and protease inhibitor cocktail), followed by three washes with RIP buffer. After proteinase K digestion, the RNA samples were extracted using RNAiso Plus (Takara, 9108Q) and then sequenced. The high-throughput sequencing was conducted by Seqhealth Technology Co., Ltd. (Wuhan, China). The raw count matrix was filtered for low expressed genes (total less than 100 counts) and normalized using the DESeq2 R package version 1.40.0, then analyzed using Tidyverse R package 1.3.0. The parameter for classifying significant enrichment was log2 (relative percentage of input) ≥1 and *P*-value <0.05. Subset of genes screened for GO analysis was performed using the clusterProfiler R package version 4.0. Visualization of analysis results and other analysis of data were performed using in R 4.0.0.

### qRT-PCR analysis

Total RNA was isolated by RNAiso Plus (Takara, 9108Q). The RNA amount was determined using NanoDrop One (Thermo Fisher Scientific). The same amount of RNA was reverse transcribed into cDNA using the PrimeScript RT reagent kit with gDNA Eraser (Takara, RR047Q). The generated cDNA was subjected to real-time PCR assay using TB Green Premix Ex Taq II (Tli RNaseH Plus) (Takara, RR820A). The relative quantities of cDNA determined by performing a comparative Ct (ΔΔCt) experiment were normalized to *GAPDH* or *U6*. For RIP qRT-PCR analysis, *U6* was used as a negative control to determine fold enrichment. The qRT-PCR primers used in this study are provided in the Supplementary Materials.

### Establishment of CRISPR/Cas9-based *CTNNB1* knock-out HEK 293T cell lines

sgRNA sequences targeting exons of the human *CTNNB1* genes were designed using an online CRISPR Design Tool (http://tools.genome-engineering.org). Oligos (forward sequence: 5′-CACCGACCCAGCGCCGTACGTCCA-3′ and reverse sequence: 5′-AAACTGGACGTACGGCGCTGGGTC-3′) were synthesized, annealed, and ligated to the Esp3I (NEB, R0374S)-digested lentiCRISPR v2 plasmid using T4 DNA ligase (Thermo, EL0014). A lentiCRISPR v2 plasmid containing sgRNA with no human genome target sequence (GTATTACTGATATTGGTGGG) (scramble) was used as the control. For the lentivirus package, in six-well plates, HEK 293T cells were transfected with CTNNB1-sgRNA LentiCRISPR v2 plasmid (800 ng) or scramble-sgRNA LentiCRISPR v2 plasmid (800 ng) and two packaging plasmids, pCMV-dR8.91 (1,200 ng) and pMD2.G (400 ng). The culture medium was replaced with maintenance medium 6 h after transfection. After an additional 42 h, the supernatant containing recombinant virus was filtered through a sterile syringe filter with a 0.45 µm pore size hydrophilic PVDF (polyvinylidene fluoride) membrane (Millipore, SLHV033R) and used to infect HEK 293T cells three subsequent times. Four days after the first transduction, cells were selected by incubating with 1 µg/mL puromycin (MedChemExpress, HY-B1743A) for another 10 days, and then a single cell was isolated by serial dilutions and allowed to expand for 2–3 weeks without puromycin. At least 10 cell clones of each transformation were further confirmed by Western blotting.

### Animal study

Five- to six-week-old C57BL/6 mice were kept in an environmentally controlled specific pathogen-free animal facility in the State Key Laboratory of Pathogens and Biosecurity (Beijing, China). Mice were divided into three groups: SFTSV + vehicle group (*n* = 10), SFTSV + NTZ group (*n* = 10), and DMEM + NTZ group (*n* = 5). For SFTSV infection, mice were treated with anti-IFNAR1 IgG (1.7 mg) by intraperitoneal injection 1 day prior to infection. Mice were intraperitoneally inoculated with 2 × 10^3^ FFU of SFTSV in 100 µL of DMEM at day 0 or the same volume of DMEM. NTZ (MedChemExpress, HY-B0217) was dissolved in 0.4% carboxymethyl-cellulose and administered by intragastric administration at a dose of 200 mg/day 1 h after SFTSV inoculation. NTZ was administered on a daily basis for 5 days, and the mice were monitored for 12 days. Prior to sacrifice, blood samples were collected via cardiac puncture from anesthetized mice and used for serum isolation. Spleen and liver samples were subsequently collected. The titration of virus was determined by the immunostaining plaque assay described above. Western blotting was used to detect CTNNB1 and SFTSV NP protein levels in spleen samples. The spleen samples were fixed in 4% formaldehyde in PBS at room temperature. The fixed tissues were paraffin-embedded, sectioned, and processed for IHC using antibodies against mouse CTNNB1 (Abcam, SP328) and SFTSV NP protein. All experiments were conducted with the approval of the Institutional Animal Care and Use Committee at the Beijing Institute of Microbiology and Epidemiology (IACUC-IME-2021-003) and were performed in accordance with the National Institutes of Health guidelines under protocols.

### Statistical analysis

All statistical analyses were performed using Microsoft Excel, Prism 9.0 software, R 4.0.0, and Image Lab 5.2.1. Data are presented as the mean ± SD. Two-tailed Student’s *t*-test, unpaired *t*-test, one-way ANOVA (analysis of variance), or two-way ANOVA were applied to determine statistical significance. The Kaplan–Meier method was adopted for animal survival analysis to generate graphs, and the survival curves were analyzed with log-rank analysis. Significance was set to *P* < 0.05 (*P < 0.05, **P < 0.01, ***P < 0.001, ****P < 0.0001), ns, not significant. The statistical information of each experiment, including the statistical methods, and number of experiments’ replicates are shown in the figure legends.

## Data Availability

The data that support the findings of this study are available from the corresponding authors upon request.

## SUPPLEMENTAL MATERIAL

Supplemental figures mbio.01688-23-s0001.docx Fig. S1-S13.

Table S1 mbio.01688-23-s0002.xlsx RIP-seq analysis showing SFTSV L protein interacted candidate genes.

Table S2 mbio.01688-23-s0003.xlsx GO-term enrichment analysis was performed on SFTSV L protein interacted genes.

Table S3 mbio.01688-23-s0004.xlsx Primers used in qRT-PCR experiments.

ASM does not own the copyrights to Supplemental Material that may be linked to, or accessed through, an article. The authors have granted ASM a non-exclusive, world-wide license to publish the Supplemental Material files. Please contact the corresponding author directly for reuse.
