# Supplementary material for: Regulation of the WNT-CTNNB1 signaling pathway by severe fever with thrombocytopenia syndrome virus in a cap-snatching manner
Source: mBio. 2023 Oct 26;14(6):e01688-23. doi: 10.1128/mbio.01688-23 (PMC10746258; doi:10.1128/mbio.01688-23)
Supplement: Supplemental figures — Fig. S1-S13. [file mbio.01688-23-s0001.docx]

**Supporting materials**

**
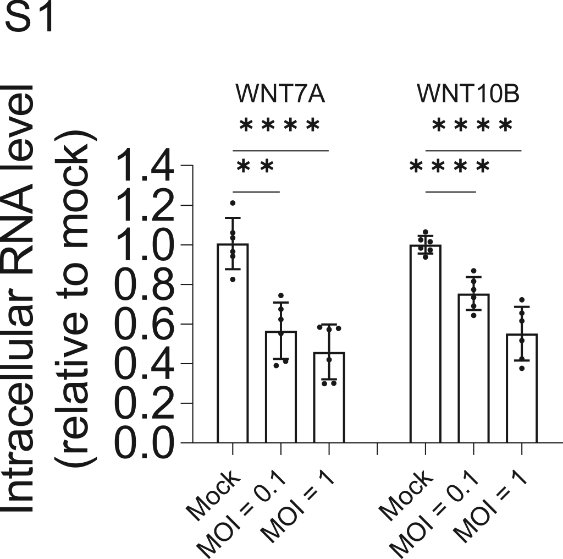
**

**Fig S1**. **Detection expression of WNT7A and WNT10B in the PBMCs infected with SFTSV**. Peripheral blood mononuclear cells (PBMCs) were isolated and collected from healthy donors. These cells were infected with SFTSV at indicated MOIs (0.1, 1) or mock. At 48 h post-infection, cells were collected and intracellular RNA was extracted. Quantitative real-time PCR (qRT-PCR) analysis of *WNT7A* and *WNT10B* normalized to *GAPDH*.

The comparison of mean values between two groups was analyzed by One-way ANOVA analyses. The experiment was performed three technical replicates.

**
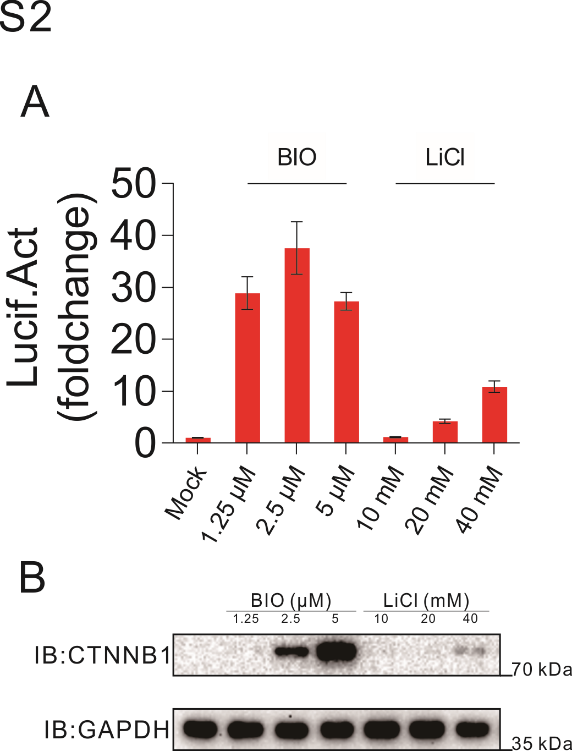
**

**Fig S2**. **The TOPFlash reporter system for the WNT-CTNNB1 signaling pathway can be activated by BIO, LiCl**. (A) In 48-well plates, HEK 293T cells were transfected with TOPFlash reporter plasmids (50 ng/well) and pRL-TK internal control luciferase reporter plasmids (50 ng/well). At 24 h post-transfection, cells were treated with BIO or LiCl at indicated concentrations. After 24 h, cells were lysed and subjected to luciferase assays to measure TOPFlash activity. (B) In 24-well plates, HEK 293T cells were treated with BIO or LiCl at indicated concentrations. After 24 h, the cells were lysed and subjected to Western blot analysis. Blots showing the accumulation of CTNNB1. GAPDH was also detected as a control for each lane.

The experiment was performed three independent replicates and any independent replicates had three technical replicates. Immunoblot was representative of three independent experiments.

**
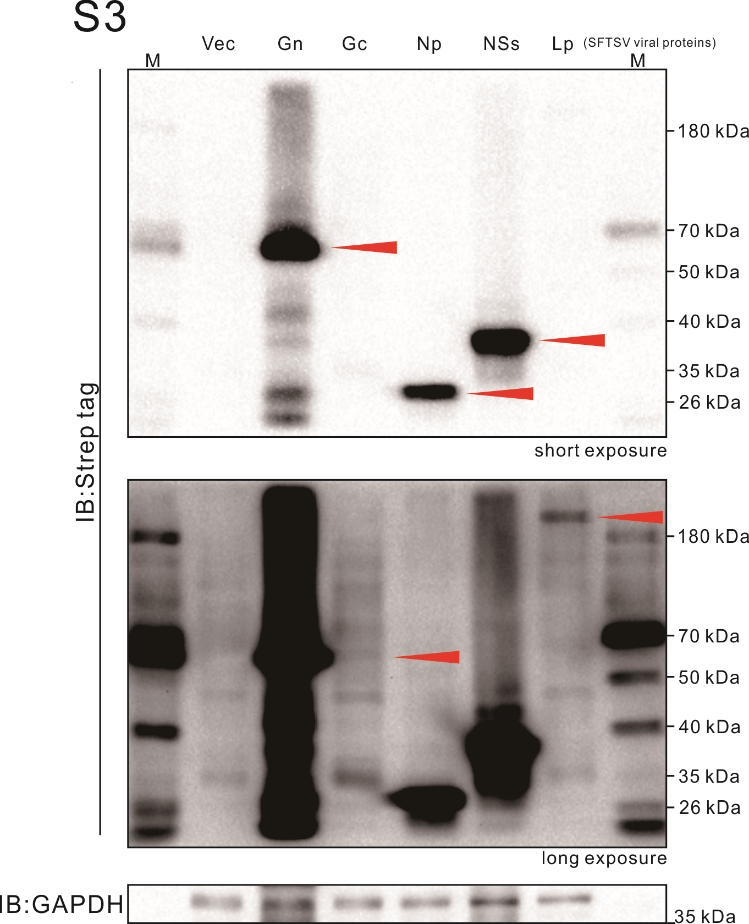
**

**Fig S3**. **Western Blots showing the expression levels of all viral proteins detected with a rabbit polyclonal antibody against the strep tag**. In 6-well plates, HEK293T cells were transfected with pCAGGS plasmids encoding SFTSV viral proteins with a C-terminal Twin-Strep-tag or a pCAGGS vector plasmid expressing only the Twin-Strep-tag as a control (1600 ng/well). At 48 h transfection, cell lysates were subjected to immunoblots with an anti-Strep tag antibody. Viral proteins were indicated by red triangles. Exposure times are reported as long exposure and short exposure. GAPDH was also detected as a control for each lane. Vec, pCAGGS vector plasmid; Gn/Gc/Np/NSs/Lp, pCAGGS plasmid encoding SFTSV Gn, Gc, NSs, N or L protein; M, marker.

Immunoblot was representative of three independent experiments. The corresponding detected antibody of the band was labeled on the left, the maker size near the band was labeled on the right.

**
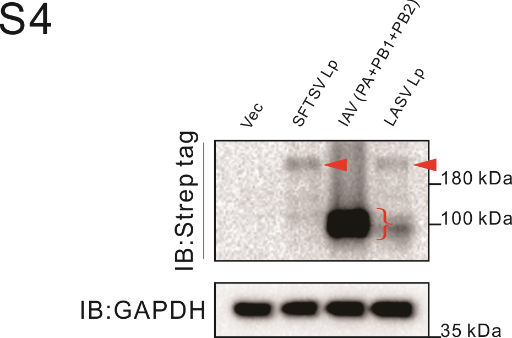
**

**Fig S4**. **Western Blots showing the expression levels of LASV L protein and IAV polymerase complex (PA, PB1 and PB2) detected with a rabbit polyclonal antibody against the strep tag**. In 6-well plates, HEK293T cells were transfected with pCAGGS plasmids encoding SFTSV L protein, LASV L protein (1500 ng/well) and IAV ploymerase complex (PA, PB1 and PB2) (500 ng/well, respectively) with a C-terminal Twin-Strep-tag or a pCAGGS vector plasmid expressing only the Twin-Strep-tag as a control (1500 ng/well). At 48 h transfection, cell lysates were subjected to immunoblots with an anti-Strep tag antibody. SFTSV and LASV L proteins were indicated by red triangles, and the red curly bracket indicated IAV PA, PB1 and PB2 proteins of similar size. GAPDH was also detected as a control for each lane. Vec, pCAGGS plasmid vector; SFTSV Lp, pCAGGS plasmid encoding SFTSV L protein; LASV Lp, pCAGGS plasmid encoding LASV L protein; IAV (PA+PB1+PB2), pCAGGS plasmid encoding IAV PA, PB1 or PB2 protein, respectively.

Immunoblot was representative of three independent experiments. The corresponding detected antibody of the band was labeled on the left, the maker size near the band was labeled on the lower right.

**
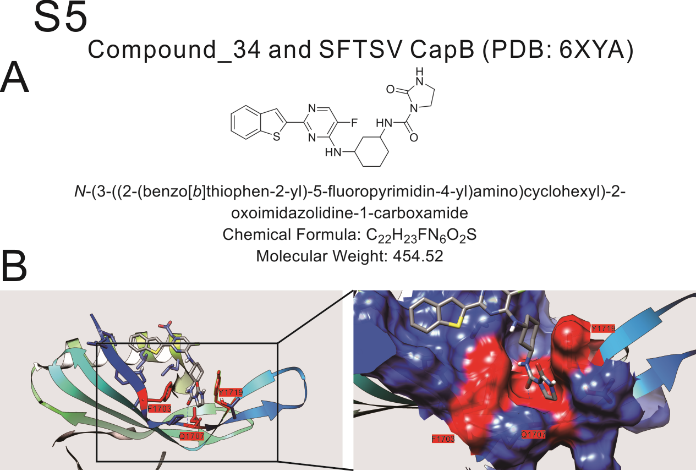
**

**Fig S5. Compound_34 molecular information and the docking pose of Compound_34 in the Cap-binding pocket of SFTSV CapB (PDB ID: 6XYA).** The chemical name, chemical formula, structural formula and molecular weight of Compound_34 (A). The docking pose of Compound_34 in the Cap-binding pocket of SFTSV CapB (PDB ID: 6XYA) (B). The compound was shown in the form of stick, and amino acids away from it (< 5Å) around the binding region were shown in the form of surface (magnified view). Important amino acid sequence information was also shown in red, and hydrogen bond interaction between compound and amino acid (Q1707) was highlighted as blue dashed lines. The molecular formula of Compound_34 was illustrated with ChemDraw 22.0.0 software, the molecular docking study of the compound and SFTSV CBD was performed using SYBYL-X 2.1.1 software, and the visualization of docking result carried out using UCSF Chimera 1.16 software.

**
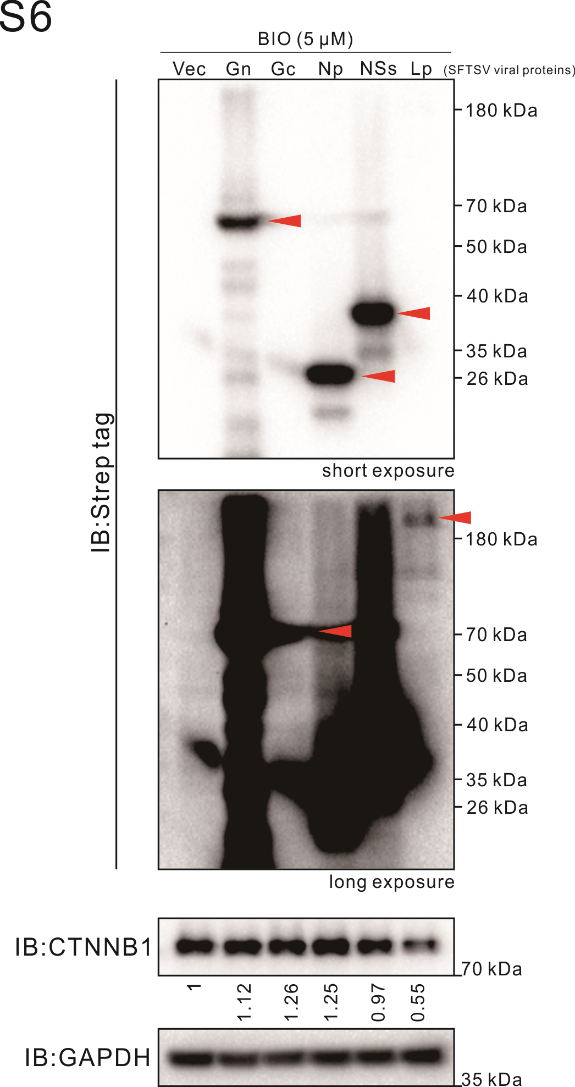
**

**Fig S6. SFTSV L protein could reduce the intracellular protein level of CTNNB1.** In 6-well plates, HEK293T cells were transfected with pCAGGS plasmids encoding SFTSV viral proteins with a C-terminal Twin-Strep-tag or a pCAGGS vector plasmid expressing only the Twin-Strep-tag as a control (1600 ng/well). At 24 h post-transfection, cells were treated (BIO, 5 µM). After 24 h, cells were collected for Western blot analysis. SFTSV viral proteins and CTNNB1 were detected. Viral proteins were indicated by red triangles. GAPDH was also detected as a control for each lane. Vec, pCAGGS vector plasmid; Gn/Gc/Np/NSs/Lp, pCAGGS plasmids encoding SFTSV Gn, Gc, NSs, N or L protein.

Immunoblot was representative of three independent experiments. The corresponding detected antibody of the band was labeled on the left, the maker size near the band was labeled on the lower right. Gray value analysis of the above bands using Image Lab. Relative quantitative results are shown below each corresponding band.

**
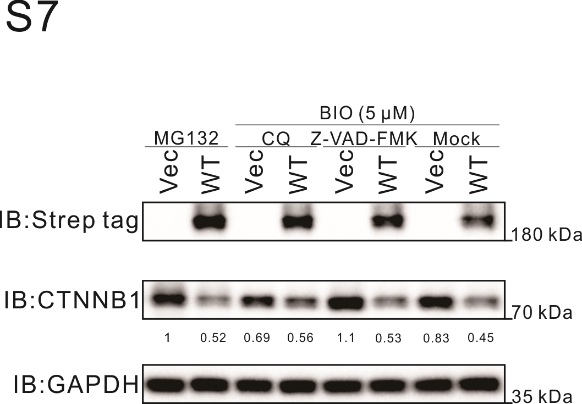
**

**Fig S7**. **Effects of protein-degradation related inhibitors on SFTSV L protein mediated reduction of CTNNB1**. In 12-well plates, HEK293T cells were transfected with pCAGGS plasmids encoding SFTSV L protein or vector. At 24 h post transfection, cells were treated with MG132 (10 µM), CQ (20 µM), Z-VAD-FMK (20 µM) or mock. All were activated with BIO (5 µM) except those treated with MG132. After 6 h, Cell lysates were subjected to immunoblots with the indicated antibodies. MG132, a potent proteasome inhibitor; CQ, Chloroquine, an autophagy and lysosome inhibitor; Z-VAD-FMK, an irreversible pan-caspase inhibitor. GAPDH was also detected as a control for each lane.

Immunoblot was representative of three independent experiments. The corresponding detected antibody of the band was labeled on the left, the maker size near the band was labeled on the lower right. Gray value analysis of the above bands using Image Lab. Relative quantitative results were shown below each corresponding band.

**
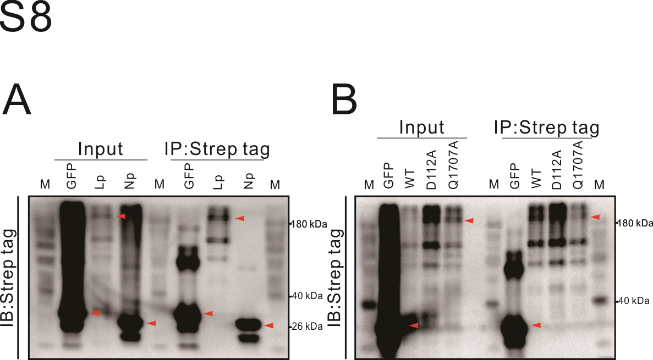
**

**Fig S8. Western blots showing protein expression levels in RIP/qRT-PCR analysis..** RIP/qRT-PCR analysis of SFTSV L protein or N protein interacting mRNAs as shown in Fig 5F-G. The cells were subjected to RNA immunoprecipitation (RIP) with MagStrep XT beads, followed by immunoblotting (A and B). Viral proteins were indicated by red triangles. GFP, SFTSV L (wide type and mutant) and N proteins were detected with anti-Strep tag antibody. GFP, pCAGGS plasmid encoding GFP; Lp/Np, pCAGGS plasmid encoding SFTSV L or N protein; M, marker.

Immunoblot was representative of three independent experiments. The corresponding detected antibody of the band was labeled on the left.

**
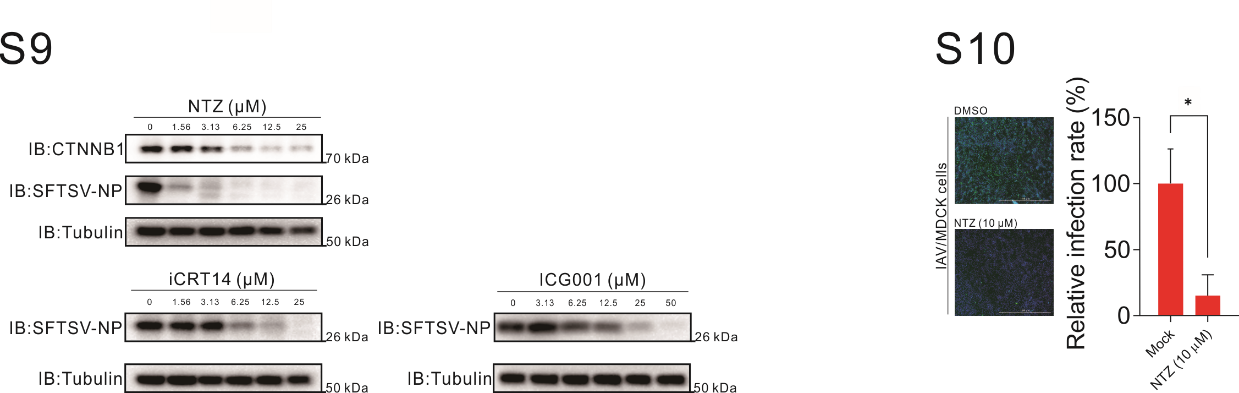
**

**Fig S9-10**. **Inhibitors of the WNT-CTNNB1 signaling pathway could inhibit SFTSV and IAV replication**. **S9**, In 24-well plates, HEK 293T cells pre-infected with SFTSV at an MOI of 0.1 were treated with a series concentration of these pathway inhibitors. After 48 h, the cells were collected and subjected to Western blot analysis. GAPDH was also detected as a control for each lane. **S10**, Immunofluorescence analysis showed that NTZ (10 µM) could effectively inhibit IAV replication in MDCK cells. In 96-well plates, MDCK cells pre-infected with IAV at an MOI of 0.01 were treated with NTZ at 10 µM. At 48 h post-infection, cells were fixed by formaldehyde and subjected to immunofluorescence analysis. Bars represent 1000 µm.

The comparison of mean values (S10) between the two groups was analyzed by Student’s analyses. All experiments were performed with three independent replicates and any independent replicates had at least two technical replicates. Immunoblot was representative of three independent experiments. The corresponding detected antibody of the band was labeled on the left, and the maker size near the band was labeled on the lower right. The concentrations of the inhibitors used were indicated above the band.

**
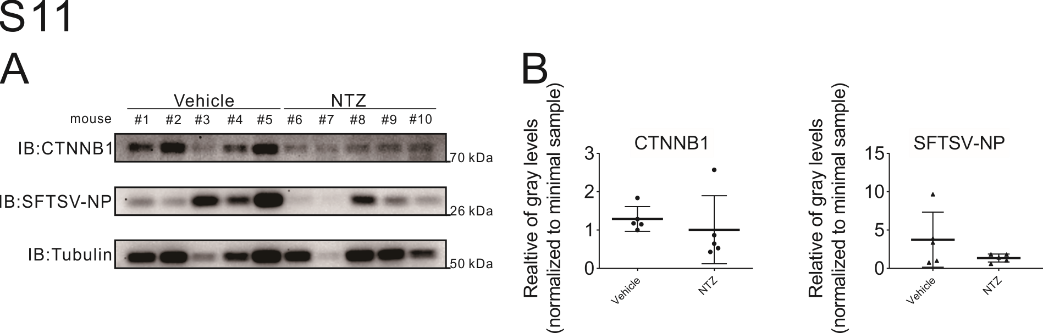
**

**Fig S11**. **Immunoblot analysis of CTNNB1 and SF-NP in spleen tissues derived from vehicle and NTZ-treated mice**. (A) Western blots detecting CTNNB1 and SFTSV NP protein levels in spleen samples. GAPDH was also detected as a control for each lane. (B) Gray value analysis of the above bands using Image Lab. GTNNB1 an SFTSV NP gray levels of all samples were normalized to minimal one. Immunoblots were representative of three technical replicates. The mouse number and group information were labeled above the band.

**
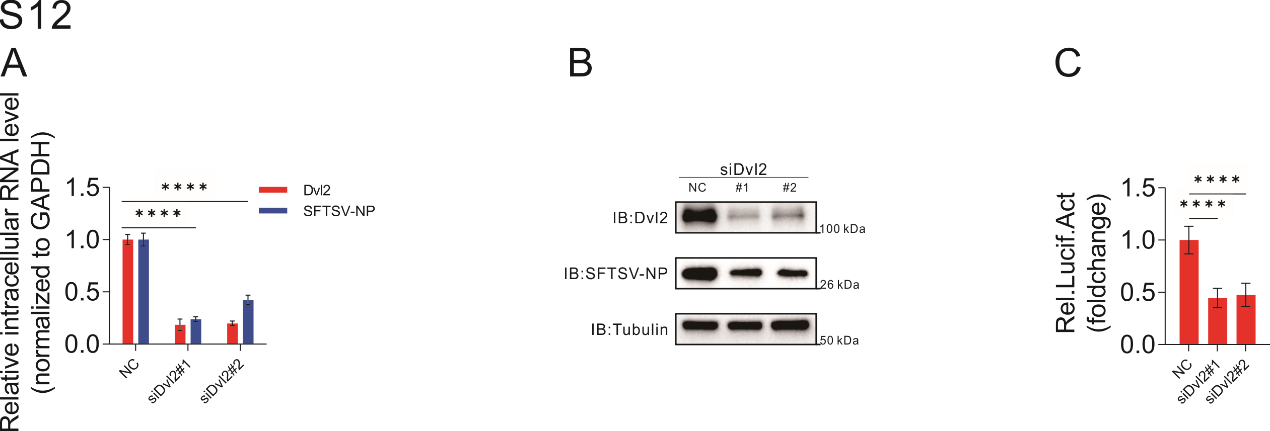
**

**Fig S12**. **Knockdown of DVL2 affected SFTSV replication in HEK293T cells.** RNAi-based knockdown of DVL2 in HEK 293T cells. (A-B) In 12-well plates, HEK 293T cells were transfected with siRNAs specifically for DVL2 (siDvl2#1 or siDvl2#2) or a control (NC) for 48 h, 40 pmol/well, and then infected with SFTSV (MOI = 0.1) for 24 h, followed by qPCR and Western blotting. (C) TOPFlash assay of RNAi-based knockdown of DVL2 in HEK293T cells. In 48-well plates, HEK 293T cells were transfected with TOPFlash reporter plasmids (50 ng/well), pRL-TK internal control luciferase reporter plasmids (50 ng/well) and siRNAs specifically for DVL2 (siDvl2#1 or siDvl2#2) or a control (NC) (20 pmol/well). At 48 h post-transfection, cells were treated with BIO (2.5 µM) or mock. After 24 h, cells were lysed and subjected to luciferase assays to measure TOPFlash activity.

The comparison of mean values (A and C) between the two groups was analyzed by One-way ANOVA or two-way ANOVA analyses. All experiments were performed with three independent replicates and any independent replicates had at least two technical replicates. Immunoblot was representative of three independent experiments. The corresponding detected antibody of the band was labeled on the left, and the maker size near the band was labeled on the lower right.

**
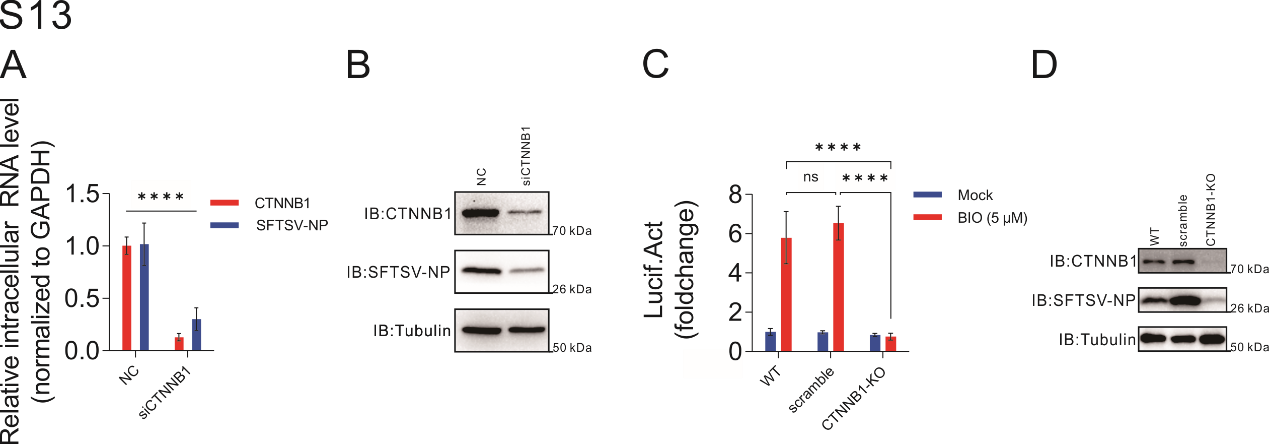
**

**Fig S13**. **Knockdown or knockout of CTNNB1 affected SFTSV replication in HEK293T cells**. (A-B) RNAi-based knockdown of CTNNB1 in HEK293T cells. In 12-well plates, HEK 293T were transfected with siRNAs specifically for CTNNB1 (siCTNNB1) or control (NC) for 48 h, 40 pmol/well, and then infected with SFTSV (MOI=0.1) for 24 h, followed by qPCR and Western blotting. (C) TOPFlash assay of CRISPR/Cas9-based CTNNB1-knockout HEK293T cell lines, untreated cells or scramble control cells. In 48-well plates, theses cell lines were transfected with TOPFlash reporter plasmids (50 ng/well) and pRL-TK internal control luciferase reporter plasmids (50 ng/well). At 24 h post-transfection, cells were treated with BIO (5 µM) or mock. After 24 h, cells were lysed and subjected to luciferase assays to measure TOPFlash activity. (D) In 24-well plates, the three cell lines were infected with SFTSV at an MOI of 1 and untreated or scramble-transfected cells were used as controls. At 48 h post-infection, the cells were collected and subjected to Western blot analysis. Tubulin was also detected as a control for each lane.

Comparison of mean values (A and C) between two groups was analyzed by One-way ANOVA or two-way ANOVA analyses. All experiments were performed three independent replicates and any independent replicates had at least two technical replicates. Immunoblots were representative of three independent experiments. The corresponding detected antibody of the band was labeled on the left, the maker size near the band was labeled on the lower right.
